# *In vitro* and *in silico* assessment of probiotic and functional properties of *Bacillus subtilis* DE111^®^

**DOI:** 10.3389/fmicb.2022.1101144

**Published:** 2023-01-13

**Authors:** Shahneela Mazhar, Ekaterina Khokhlova, Joan Colom, Annie Simon, John Deaton, Kieran Rea

**Affiliations:** ^1^Deerland Ireland R&D Ltd., ADM, Food Science Building, University College Cork, Cork, Ireland; ^2^Deerland Probiotics and Enzymes, ADM, Kennesaw, GA, United States

**Keywords:** *Bacillus subtilis*, probiotics, antimicrobial, antioxidant, metabolism, functional foods, *in vitro*, *in silico*

## Abstract

*Bacillus subtilis* DE111^®^ is a safe, well-tolerated commercially available spore-forming probiotic that has been clinically shown to support a healthy gut microbiome, and to promote digestive and immune health in both adults and children. Recently it was shown that this spore-forming probiotic was capable of germinating in the gastrointestinal tract as early as 3 h after ingestion. However, a better understanding of the mechanisms involved in the efficacy of DE111^®^ is required. Therefore, the present investigation was undertaken to elucidate the functional properties of DE111^®^ through employing a combination of *in vitro* functional assays and genome analysis. DE111^®^ genome mining revealed the presence of several genes encoding acid and stress tolerance mechanisms in addition to adhesion proteins required to survive and colonize harsh gastrointestinal environment including multi subunit ATPases, arginine deiminase (ADI) pathway genes (*argBDR*), stress (GroES/GroEL and DnaK/DnaJ) and extracellular polymeric substances (EPS) biosynthesis genes (*pgsBCA*). DE111^®^ harbors several genes encoding enzymes involved in the metabolism of dietary molecules (protease, lipases, and carbohyrolases), antioxidant activity and genes associated with the synthesis of several B-vitamins (thiamine, riboflavin, pyridoxin, biotin, and folate), vitamin K2 (menaquinone) and seven amino acids including five essential amino acids (threonine, tryptophan, methionine, leucine, and lysine). Furthermore, a combined *in silico* analysis of bacteriocin producing genes with *in vitro* analysis highlighted a broad antagonistic activity of DE111^®^ toward numerous urinary tract, intestinal, and skin pathogens. Enzymatic activities included proteases, peptidases, esterase’s, and carbohydrate metabolism coupled with metabolomic analysis of DE111^®^ fermented ultra-high temperature milk, revealed a high release of amino acids and beneficial short chain fatty acids (SCFAs). Together, this study demonstrates the genetic and phenotypic ability of DE111^®^ for surviving harsh gastric transit and conferring health benefits to the host, in particular its efficacy in the metabolism of dietary molecules, and its potential to generate beneficial SCFAs, casein-derived bioactive peptides, as well as its high antioxidant and antimicrobial potential. Thus, supporting the use of DE111^®^ as a nutrient supplement and its pottential use in the preparation of functional foods.

## 1. Introduction

The demand for probiotics and probiotics-enriched functional foods has been rapidly growing, with the increased consumer awareness of the health-promoting benefits of the microbes that inhabit our gut. The World Health Organization defines probiotics as “live microorganisms that, when administered in adequate amounts, confer a health benefit on the host” (Hill et al., 2014). A limiting factor of many probiotics with potential functional health benefits is that they can have issues with stability and survivability during processing, preservation, treatments, storage, and their passage through the gastrointestinal tract (GI; Siciliano et al., 2021). However, spore-based probiotics due to their very nature have innate properties to help them overcome some of these limitations (Ramlucken et al., 2021). Spore-forming microbes, including those from the *Bacillus* genera can survive in extreme environmental conditions including heat and certain chemicals, enabling long-term survival in situations that could kill other types of vegetative bacteria (Lee et al., 2019). Recent studies have also demonstrated that spores of *Bacillus* probiotics germinate, grow, and resporulate in the GI tract (Casula and Cutting, 2002; Colom et al., 2021). The commercial *Bacillus* probiotic strains in use are *Bacillus cereus, Bacillus clausii*, *Bacillus coagulans*, *Bacillus indicus*, *Bacillus licheniformis*, *Bacillus polyfermenticus*, *Bacillus pumilus*, and *Bacillus subtilis* (Lee et al., 2019; Saggese et al., 2021). Several studies have shown that members of these *Bacillus* species can proliferate in the intestines and produce antimicrobial substances, including bacteriocin, short-chain fatty acids, and organic acids (Duc et al., 2004; Efremenkova et al., 2019; Chau et al., 2020). These antimicrobials and antiadhesion effects against pathogen strains contribute to modulating GI disorders. Additionally, *in silico* analysis combined with animal and human studies have demonstrated *Bacillus* probiotic strain’s potential to participate in immuno-modulatory roles, anticancer effects, lowering cholesterol, and enhancing vitamin production (Lee et al., 2015; Kapse et al., 2019; Marzorati et al., 2020; Trotter et al., 2020).

*Bacillus subtilis* is a Gram-positive, spore-forming, rod-shaped facultative anaerobic bacterium (Olmos, 2014). Bss-19, DE111^®^, and SG188 strains are members of this species that have been considered as Generally Recognized as Safe (GRAS) substance (Gras notice number; 969, 831, and 905, respectively), under the conditions of their intended use in conventional foods and as a probiotic food ingredient. In general, *B. subtilis* has a long consumption history and is an established ingredient in the preparation of several traditional fermented foods, such as the fermented foods from soybeans and locust beans in Asian and West African countries, respectively. *B. subtilis* strains have been tested in several randomized, placebo-controlled clinical studies investigating the impact of these probiotic strains in several metabolic and digestive conditions including abdominal discomfort, immunity, bone mineral density, antibiotic associated diarrhea, gastrointestinal viability, general wellness, microbiome change and epidermal symptoms (Guo et al., 2016). From these studies the findings suggest that *B. subtilis* can (a) secrete a variety of enzymes to assist in the degradation and digestion of dietary lipids, proteins, and carbohydrates (b) promote growth performance and intestinal morphology, and (c) positively affect host health status by synthesizing different antimicrobial compounds modifying the host-associated microbial community and stimulating innate immunity (Zokaeifar et al., 2012; Elshaghabee et al., 2017; Ringel-Kulka et al., 2017; Mehta et al., 2020).

*Bacillus subtilis* DE111^®^ is a safe, commercially available probiotic that has been shown to be well tolerated, supporting a healthy gut microbiome and promoting digestive and immune health in both adults and children (Townsend et al., 2018; Labellarte et al., 2019; Paytuví-Gallart et al., 2020; Slivnik et al., 2020; Toohey et al., 2020; Colom et al., 2022). Clinical studies have shown DE111^®^ to alleviate occasional constipation or diarrhea in healthy adults, while regulating blood lipid levels and suppressing gut and peripheral inflammation (Cuentas et al., 2017; Trotter et al., 2020; Freedman et al., 2021). In adult athletes, DE111^®^ consumption reduced circulating TNF-α levels in blood and enhanced body composition in females when combined with post-workout nutrition (Toohey et al., 2020; Townsend et al., 2021). In pediatric cohort (children aged between 2 and 6 years) DE111^®^ was well tolerated and was associated with positively modulating the gut microbiome (Paytuví-Gallart et al., 2020). Additionally, in healthy adult population DE111^®^ daily intake has illustrated significant reduction in total cholesterol and non-HDL cholesterol (Trotter et al., 2020).

To further delineate the mechanisms involved in the efficacy of DE111^®^ whole genome sequence analysis was used to mine the genome for genes encoding antimicrobial peptides, digestive enzymes, amino acids, and vitamin production. This computer-based approach was complemented with assays for antimicrobial activity, enzymatic activity, and metabolite production in various cell-based models. The functional capabilities of this probiotic strain were further interrogated including temperature stability, adhesion to mucous and non-mucous secreting intestinal cell lines, and antioxidant activity. To understand the overall probiotic potential, the genetic repertoire of DE111^®^ required for surviving harsh gastric transit and conferring health benefits was coupled with the phenotypic, enzymatic and metabolomics data generated by gas chromatography–mass spectrometry (GC-MS) and other methodologies.

## 2. Materials and methods

### 2.1. Bacterial strains and culture conditions

*Bacillus subtilis* DE111^®^ was cultivated at 37°C for 24 h in Tryptic Soy Broth (TSB; Becton, Dickinson and Company, Berkshire, England). Indicator strains employed to determine the antimicrobial activity of *B. subtilis* DE111^®^ were either purchased from the American Type Culture Collection (ATCC, Middlesex, United Kingdom) or Deutsche Sammlung von Mikroorganismen und Zellkulturen GmbH (DSMZ, Braunschweig, Germany). These are listed in Table 1. All of these strains except *Cutibacterium acnes* and *Gardernella vaginalis* were cultivated at 37°C for 24 h in TSB (Becton, Dickinson and Company, Berkshire, England). *Cutibacterium acnes* was grown in Brain heart infusion medium (BHI) (Merck, Darmstadt, Germany), and *G. vaginalis* was cultivated in New York City (NYC) medium, both were incubated at 37°C under anaerobic conditions. In addition, other control strains employed for comparative analysis were *Lactobacillus rhamnosus* (ATCC 53103) cultivated at 37°C for 24 h in TSB (Becton, Dickinson and Company, Berkshire, England), and *Lactobacillus fermentum* ME*-*3 cultivated at 37°C for 24 h in De Man, Rogosa and Sharpe broth (MRS; Merck, Darmstadt, Germany).

**Table 1:** Indicator strains used in this study.

Bacterial Species	Strain designation
*Salmonella enteritidis*	ATCC13076
*Pseudomonas aeruginosa*	DSM3227
*Yersinia enterocolitica*	DSM4780
*Staphylococcus warneri*	DSM20316
*Escherichia coli*	ATCC25922
*Staphylococcus epidermidis*	DSM20044
*Listeria monocytogenes*	DSM20600
*Shigella flexnerii*	DSM4782
*Candida albicans*	DSM3454
*Cutibacterium acnes*	DSM1897
*Staphylococcus pseudintermedius*	DSM21284
*Staphylococcus saprophiticus*	DSM20229
*Staphylococcus aureus*	DSM1104
*Staphylococcus aureus*	DSM17091
*Bacillus cereus*	DSM31
*Corynebacterium flavescens*	DSM20296
*Acinetobacter baumannii*	DSM30007
*Enterococcus faecalis*	DSM20478
*Streptococcus agalactiae*	DSM2134
*Streptococcus pyogenes*	DSM20565
*Gardernella vaginalis*	DSM4944
*Campilobacter jejuni*	DSM4688

### 2.2. Temperature stability

Temperature stability of DE111^®^ spores (5 × 10^9^ CFU) were determined at different pasteurization temperatures and was studied in 1× PBS, Oat milk (Tesco, Ireland) and 5% Apple juice (100%, Lidl, Ireland). The pH of 1× PBS suspension was 7.59 and the pH of oat milk was 7.61. Apple juice was prepared using deionized water, yielding 5% apple juice with a pH of 4.18. Samples were collected at four time points during the pasteurization (0, 0.5, 1, and 3 min) at 45, 75, and 90°C.

### 2.3. Antimicrobial activity

A modification of a double layered agar method was used for the determination of antimicrobial activity of DE111^®^ against indicator strains summarized in Table 1 (Stachurska et al., 2021). TSA was used as a base layer and was spotted with five microliters of 1:10 diluted stationary phase DE111^®^ culture (approximately 10^8^ CFU/ml). The spots were then left to dry, and the plates were incubated at 37°C for 18 h. Five milliliters of molten TSA (0.3–0.5% agar) inoculated with 100 μl of 1:100 diluted stationary phase of indicator strains suspension (approximately 10^9^ CFU/ml) was used to overlay the plates. The plates were incubated at 37°C for 18 h and the inhibition zones against the test strains were observed. The antimicrobial activity against *C. acnes* and *G. vaginalis* was characterized by using 5 ml of a 1:10 diluted stationary phase *C. acnes* culture poured onto BHI plates with grown *B. subtilis* DE111^®^ spots. After spreading the *C. acnes* suspension on to the plate, excess liquid was removed using a micropipette and the plates were left to dry. Similarly, 5 ml of freshly prepared “soft” NYC (0.5% agar) inoculated with 0.5 ml of overnight culture *G. vaginalis* overlayed TSA plates with grown *B. subtilis* DE111^®^ spots. These plates were incubated at 37°C under anaerobic conditions for 18 h and the inhibition zones were observed.

### 2.4. Antioxidant activity

The total antioxidant capacity of *B. subtilis* DE111^®^ was compared with *L. rhamnosus* (ATCC 53103) using a commercial total Antioxidant Capacity assay kit (MAK187, Merk, Darmstadt, Germany). Components of the kit were prepared according to manufacturer’s instructions. A 100 μl of the stationary phase culture *B. subtilis* DE111^®^ and *L. rhamnosus* (ATCC 53103; approximately 10^9^ CFU/ml) were transferred to 50 ml fresh TSB media in 250 ml conical flasks. The cultures were incubated overnight at 37°C, 170 RPM. The cultures were serial diluted to dilution factor of 10^6^ and spread plated on TSA plates. The plates were incubated at 37°C overnight. After incubation, counts were recorded, and the cultures were normalized using 1× PBS to perform the assay with same cell counts. The tubes were centrifuged at 4,380 × *g* for 15 min. Supernatants were discarded and the pellets were washed three times with 50 ml 1× PBS. After the wash, the pellets were weighed and resuspended in 3 ml 1× PBS and vortexed vigorously. The suspension was transferred to two fresh beaded tubes with 1.5 ml each. *B. subtilis* DE111^®^ and *L. rhamnosus* (ATCC 53103) were homogenized at 3,500 rpm for 30 s using Beadbug homogenizer (Benchmark Scientific, United States). The tubes were placed in ice for 1 min between cycles. The process was repeated three times. The homogenized samples were centrifuged at 9,000 × *g* for 15 min and the supernatants were recovered and stored at −20°C or assayed directly. 1× PBS was set as control and the sample values were subtracted from the values of control. A 20 μl of sample supernatant was brought to a final volume of 100 μl by adding 80 μl ultrapure water. Using 100 μl of supernatant and 100 μl of Cu^2+^ Working Solution were mixed in 96-well microplate (Sarstedt, Wexford, Ireland) and incubated for 90 min at room temperature. Absorbance was measured at 570 nm (A_570_) on a reader (Multiskan FC microplate photometer, Thermofisher, Ireland). A set of Trolox Standard were prepared from 0 mM to 1 mM and their activity was measured in a similar fashion. The assay gives antioxidant capacity in Trolox equivalents (ranging from 4 to 20 nmole/well).

### 2.5. Adhesion to HT-29 and HT-29-MTX cell lines

Human Colorectal Adenocarcinoma Cell Line HT-29 and mucous-secreting cell line HT-29-MTX (purchased from ATCC) were propagated using DMEM low glucose medium supplemented with 10% Fetal Bovine Serum, 100 U/ml penicillin, and 100 μg/ml streptomycin in a 5% CO_2_ atmosphere at 37°C. Cells were seeded onto 24-well plates at a density 5 × 10^5^ cell/well and cultured for 21–28 days to complete maturation. Media was replaced every 2–3 days. Prior to experiment cells were washed twice with 0.5 ml DPBS, and 0.4 ml of full media without antibiotics were added to each well. Stationary phase culture *B. subtilis* DE111^®^ were centrifuged for 10 min at 4,000 × *g*. Supernatants were removed, and bacteria were washed once with 10 ml serum-free DMEM. Ten milliliters of serum-free DMEM were added to the pellet. One hundred microliter of bacterial suspension in DMEM were added to HT-29 and HT-29-MTX cells, mixed by gentle swirl and incubated for 3.5 h in the CO_2_ incubator (5% CO_2_ atmosphere at 37°C). Control wells not containing mammalian cells were prepared and incubated in parallel in the same manner (0.1 ml of bacterial suspension plus 0.4 ml of full DMEM medium). Bacterial strain’s ability to adhere was assessed at 37°C. Upon incubation with bacterial strains HT-29 and HT-29-MTX cells were washed four times with 0.5 ml DPBS. After that, 100 μl of Trypsin/EDTA solution were added to the wells and incubated for 20 min with gentle shaking (~100 rpm) at 37°C to detach the bacterial cells from the mammalian cells. Nine hundred microliters of PBS were added to each well, contents of the wells were transferred into 1.5 ml microcentrifuge tubes with scrapping and subjected to vigorous shaking for 30 s. Serial dilutions were prepared in PBS and plated onto TSA agar or MRS agar (*L. fermentum*). Plates were incubated at 37°C for 20–24 h prior to counting colonies. Cell concentrations for the late logarithmic/stationary phase for bacteria were determined prior to adhesion experiments to ensure the recommended multiplicity of infection (MOI, 1:10–1:100 mammalian cells: bacterial cells), 3.0–6.0×10^8^ CFU/ml. All tests were performed in two biological replicates with three technical replicates per assay. The results are expressed as means ± SD.

### 2.6. Qualitative and quantitative analysis of the proteolytic activity

#### 2.6.1. Qualitative analysis of proteolytic activity

To evaluate the proteolytic activity of DE111^®^, reconstituted skim milk (RSM) agar was prepared from skim milk powder (Sigma-Aldrich, Wicklow, Ireland) at 10% (w/v) and agar (Agar; Sigma-Aldrich, Wicklow, Ireland) at 1.5% (w/v). The inoculated plates were then incubated for 24 h at 37°C. Additionally, 30 μl of the test strain overnight were inoculated in the center of the RSM plate and air dried before incubation. The inoculated plates were then incubated for 24 and 48 h at 37°C.

#### 2.6.2. Quantitative analysis of the proteolytic activity

Cell envelope proteinase activity was determined using a modification of the method previously described by Mazhar et al. (2020), which is based on the EnzCheck^®^ kit Green Fluorescence E-6638 (Molecular Probes, Eugene, OR, United States). DE111^®^ was grown in 50 ml of TSB for 24 h at 37°C. *L. rhamnosus* (ATCC 53103) was used as a comparator and was propagated in 50 ml of TSB for 24 h at 37°C with shaking. Cells were centrifuged (4,000 × *g*, 10 min, 4°C), and washed three times with 1× PBS. Components of the kit were prepared according to manufacturer’s instructions; 100 μl of cell suspension and 100 μl of prepared BODIPY^®^ FL casein solution were mixed in 96-well microplate (Sarstedt, Wexford, Ireland) and incubated for 24 h at 37°C. Fluorescence (Ex/Em 505/513 nm) was measured on a Synergy 2 reader (Bio-Tek Multi Detection Plate Reader, Winooski, VT, United States), using optimal filters: 485/20 nm for extinction and 528/20 nm for emission. A proteinase K solution (1 μg ml^−1^) was used as a positive control. A set of trypsin standards from 0 to 50 μg ml^−1^ were prepared and their activity was measured in a similar fashion. The arbitrary fluorescence units for DE111^®^ were converted and expressed as trypsin equivalents (0–50 μg ml^−1^).

### 2.7. Semi-quantitative assay for carbohydrate fermentation and hydrolytic activities (API ZYM)

A range of hydrolytic activities were determined calorimetrically on 19 naphtyl substrates using the API-ZYM kit system (BioMérieux, Hampshire, United Kingdom) and the ability of DE111^®^ to utilize various carbon sources was investigated with API 50CH (BioMérieux, Hampshire, United Kingdom) according to the manufacturer’s instructions.

### 2.8. FAA and SCFA analysis by gas chromatography-mass spectrometry (GC-MS)

One milliliter of DE111^®^ (10^8^ CFU/ml) was used to inoculate 50 ml of commercial ultra-high-temperature (UHT) Milk (Indomilk, Semarang, Central Java, Indonesia). The culture was incubated at 37°C for 48 h with shaking. After 48 h incubation, 5 ml of the samples were subjected to pH and cell count analysis and remaining sample volume was stored at −80°C. Control was uninoculated UHT milk. Mass spectrometry analysis was carried out by MS-Omics as follows. Samples were acidified using hydrochloride acid, and deuterium labeled internal standards where added. All samples were analyzed in a randomized order. Analysis was performed using a high polarity column (Zebron™ ZB-FFAP, GC Cap. Column 30 m × 0.25 mm × 0.25 μm) installed in a GC (7890B, Agilent) coupled with a quadropole detector (5977B, Agilent). The system was controlled by ChemStation (Agilent). Raw data was converted to netCDF format using Chemstation (Agilent), before the data was imported and processed in Matlab R2014b (Mathworks, Inc.) using the PARADISe software described by Johnsen et al. (2017).

### 2.9. Genomic analysis

The genome sequence data of DE111^®^ was retrieved from NCBI (GenBank accession number; CP013984.1). The subsystem distribution of DE111^®^ was analyzed using the RAST server and visualized with the SEED server (Overbeek et al., 2014). Secondary metabolite clusters were identified with antiSMASH 6.1 (Weber et al., 2015) or direct blasting. Meanwhile, BLAST genome mapping was conducted using CGView Server (Grant and Stothard, 2008).

## 3. Results

### 3.1. General genomic features of DE111^®^

**Figure 1:**
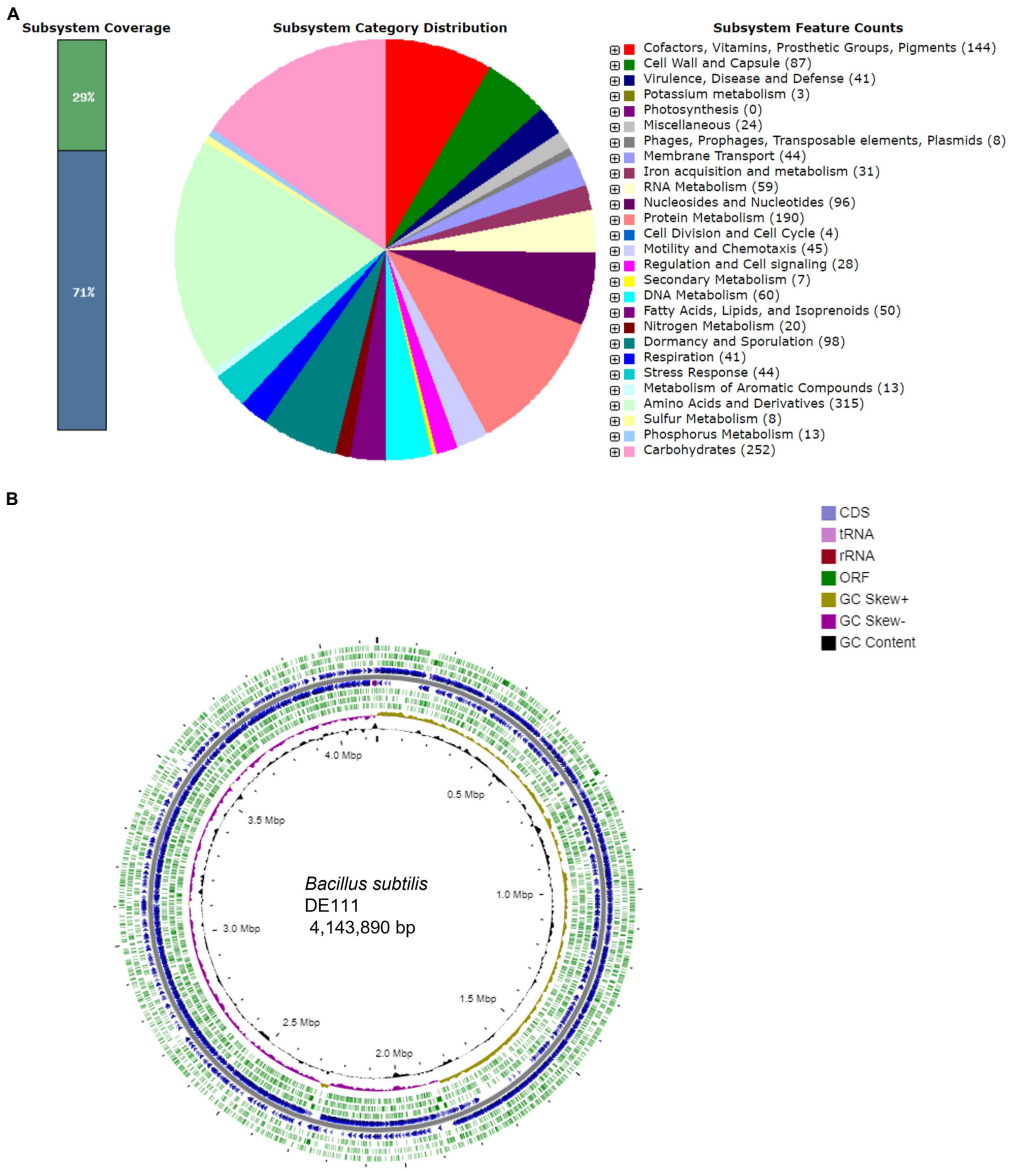
**(A)** Subsystem distribution of *Bacillus subtilis* DE111^®^ strain (based on RAST annotation server). In subsystem coverage, 29% is indicated in subsystem and 71% is not in the subsystem. The most genome ontology subsystems are amino acids and derivatives which are accounted for 315. **(B)** Genome information of *Bacillus subtilis* DE111^®^. Circles display (from the inside): Circle 1 represents GC content (black). Circle 2 represents GC skew (GC skew +: gold, GC skew −: purple). Circle 3 represents ORF’s (ORF’s − strand: green). Circle 4 represents predicted CDS (blue) and circle 5 represents ORF’s (ORF’s + strand: green). Gene GC skew (G − C/G + C), 5. rRNA (red) and tRNA (pink).

The 4.14 Mb genome of *B. subtilis* DE111^®^ featured GC content of 44.8% with 4,308 protein-coding genes and 81 RNA genes predicted using RAST annotation (Figure 1A). The subsystem distribution of *B. subtilis* DE111^®^ was analyzed using the RAST server and visualized with the SEED server. RAST server identified protein-encoding, rRNA, and tRNA genes; assigned functions to the genes, and predicted which subsystems were represented in the genome. Majority of the protein-coding genes (65.7%) were assigned with a putative function, while those remaining were annotated as hypothetical proteins. The functional classification exhibited that amino acids and derivative had a larger percentage in *B. subtilis* DE111^®^ subsystem (315). In addition to amino acids and their derivatives, carbohydrates, cofactors, vitamins, prosthetic groups, and pigments also possessed a large percentage of the subsystem (Figure 1A). The genomics information of DE111^®^ is presented in a circular model using CG view Server (Figure 1B).

### 3.2. Functional annotation of the genome unravels probiotic potential of DE111^®^

For the probiotic strains to be effective, it requires to display a set of mechanisms to overcome the adverse situations encountered through the human GIT and transiently colonize this competitive environment. This section gives an overview of the mechanisms of tolerance and defense presents in DE111^®^, enabling this microorganism to surmount these difficulties. Overall, the genome of DE111^®^ was mined for an arsenal of marker genes involved in gastric stress tolerance, adhesion, aggregation, competitive exclusion of pathogens and biosynthesis of essential vitamins and amino acids.

#### 3.2.1. Genomic insights into survival of DE111^®^ in the harsh gastric transit

One of the important criteria for any probiotic strain is its ability to survive the harsh conditions that are prevalent in the gastric tract, such as low pH and bile salt concentrations of minimum 0.5% (Ruiz et al., 2011; Kapse et al., 2019). Genomic analysis of DE111^®^ revealed the presence of a vast number of marker genes associated with acid and bile tolerance (Table 2). The first mechanism observed was F1–F10 membrane ATPase associated with regulation of cytoplasmic pH by hydrolyzing ATP to pump out H+ from cells, thereby maintaining pH homeostasis (Ryan et al., 2008; Ruiz et al., 2011). This system is enhanced by the lactate dehydrogenase (LDH) activity, which increases ATP production as a consequence of catalyzing the conversion of lactate to pyruvate, resulting in increased acid tolerance in DE111^®^. DE111^®^ genome also contained arginine/ornithine antiporter ArcD, Ornithine carbamoyl transferase and Arginine pathway regulatory protein ArgR, repressor of arg regulon, suggesting the strain’s ability to produce alkaline compounds to neutralize internal pH and acclimatize to gastric environment (Duport et al., 2016). Additionally, all genes previously reported to be involved in *B. subtilis* cell acid stress response, such as alanine dehydrogenase (ald), succinate-semialdehyde dehydrogenase (gabD), and several putative formate dehydrogenases (fdhD, yjgC, yrhE, and yrhG), where found in DE111^®^ genome (Desriac et al., 2013). Other proteins such as Sodium–hydrogen (Na+/H+) antiporters that play a major role in pH and Na + homeostasis of cells and genes encoding Glucose-6-phosphate isomerase, associated to be involved in acid tolerance as an acid-shock protein were also identified (Padan et al., 2001; Oliveira et al., 2017). Presence of genes encoding components of the Phosphotransferase system (PTS), cellobiose-specific IIC component, ATP-dependent Clp protease, ATP-binding subunit, 2, 3-bisphosphoglycerate-independent phosphoglycerate mutase, GTP pyrophosphokinase and Pyruvate kinase were also found in DE111^®^. These genes were reported to be differentially expressed in *Lactococcus* species cells cultivated under low and optimum pH (5.1–6.5; Oliveira et al., 2017). Multiple genes encoding universal stress protein UspA, which are important in oxidative and acid stress resistance in bacteria were also detected in DE111^®^, further proving the robustness of DE111^®^ to survive the acidic conditions of gut for a prolonged period (Seifart Gomes et al., 2011).

**Table 2:** Putative genes involved in gastric stress tolerance in DE111^®^ genome.

Stress response	Product	GenBank accession number
Acid tolerance	ATP synthase subunit a	AMA54196.1
	ATP synthase subunit b	AMA54194.1
		AMA54198.1
	ATP synthase Gamma chain	AMA54195.1
	ATP synthase delta chain	AMA54197.1
	ATP synthase Epsilon chain	AMA54193.1
	Na+/H+ antiporter	AMA51664.1
		AMA51856.1
	l-lactate dehydrogenase	AMA51019.1
	Alanine dehydrogenase	AMA53701.1
	Succinate-semialdehyde	AMA51109.1
	Formate dehydrogenase	AMA53196.1
	PTS system Cellobiose specific IIA subunit	AMA54365.1
	PTS system Cellobiose specific IIB subunit	AMA54366.1
		AMA54367.1
	ATP-dependent Clp protease ATP-binding subunit	AMA52061.1
		AMA50811.1
	Glucose-6-phosphate isomerase	AMA53646.1
	GTP pyrophosphokinase	AMA54355.1
	Pyruvate kinase	AMA53443.1
Acid/Bile tolerance	Arginine/ornithine antiporter ArcD	AMA53843.1
	Ornithine carbamoyltransferase	AMA51815.1
	2,3-bisphosphoglycerate-independent phosphoglycerate mutase	AMA53897.1
	arginine repressor ArgR	AMA52950.1
General stress resistance	Universal stress protein	AMA54434.1
		AMA51665.1
Bile tolerance	Glucosamine-6-phosphate deaminase	AMA50953.1
		AMA54000.1
	Chaperone protein dnaK	AMA53072.1
	Chaperone protein dnaJ	AMA53071.1
	Chaperone GroEL	AMA51311.1
	Chaperone GroES	AMA51310.1
	CTP synthase pyrG	AMA54227.1

All data presented is generated from this study.

Bile salts are surface-active, amphipathic molecules with a potent antimicrobial activity, and they act as detergents that disrupt biological membranes (Oliveira et al., 2017). In this study, several genes that were reported to confer bile resistance in other species were identified (Oliveira et al., 2017; Kapse et al., 2019). Particularly genes encoding DnaK and Enolase, these are plasminogen receptors involved in bile modulation during intestinal colonization (Oliveira et al., 2017; Table 2). Additionally, GroEL, GroES, DnaK and DnaJ chaperones, known to shield against stress imposed intracellular protein aggregation were identified in DE111^®^ genome (Azcarate-Peril et al., 2008).

Genomic determinants of sporulation in *B. subtilis* have been investigated in this study. As bacterial spores offer advantages over non-spore forming bacteria, as spores have a higher survival rate during the acidic stomach passage and better stability during the processing and storage of the food products (Bader et al., 2012). Studies have reported a minimal set of 60 genes that are essential for sporulation in this species involved in the pre-septation (Stage 0), post-septation (Stage II), post-engulfment (Stage III-VI), spore coat formation and germination (Galperin et al., 2012). A total of 74 genes involved in different phases of sporulation in DE111^®^ genome were identified (Supplementary Table S1). Overall, all stages of sporulation proteins were detected in DE111^®^ (Supplementary Table S1). At the genomic level, the presence of these genes highlights the potential of acid and bile resistant property of DE111^®^.

Adhesion to intestinal epithelium is one of the important probiotic criteria as adherence prolongs the residence of bacteria in the gut. Adhesion ability of the probiotic bacteria to the host would assist transient colonization that would help to promote immunomodulatory effects, as well as stimulate gut barrier and metabolic functions (Monteagudo-Mera et al., 2019). Based on literature data, various proteins associated with adhesion and aggregation, were predicted and are summarized in Supplementary Table S2. DE111^®^ harbors a total of 14 genes, putatively coding for adhesion-related proteins. These include chitin binding protein, enolases and γ-PGA which are regarded as common adherence factors found in *Bacillus* species (Marvasi et al., 2010; Qin et al., 2020).

#### 3.2.2. Presence of proteases, esterase’s, and carbohydrate metabolizing enzymes in DE111^®^ conferring adaptation to host nutritional environment

Genome analysis of DE111^®^ have revealed a presence of several transporters and enzymes involved in the metabolism of numerous dietary carbohydrates, proteins, and fats. Most of these dietary carbohydrates escape degradation in the upper parts of the intestine, many of which are plant-derived oligo- and polysaccharides (Pokusaeva et al., 2011). PTS transporters and enzymes involved in the conversion and metabolism of several monosaccharides, disaccharides, oligosaccharides, and polysaccharides have been identified, suggesting extensive ability of DE111^®^ to utilize wide-ranging carbohydrates from different niches such as oral cavity and GI tract (Table 3). Additionally, DE111^®^ genome encoded several peptidases, peptide transporters, lipids, and esterase’s, suggesting the efficiency of DE111^®^ to process and recover amino acids and lipids from nutritionally rich environmental sources.

**Table 3:** Distribution of transporters and enzymes in DE111^®^ that are potentially responsible for carbohydrate, protein, and lipid metabolism.

Subsystem	Functional category	Suggested gene name	Description/function	Protein accession number
*Carbohydrate metabolism*				
Monosaccharide	Ribose metabolism	*rbsA*	Periplasmic ribose binding protein	AMA54097.1	*rbsB*	d-Ribose ABC transporter substrate-binding protein	AMA54099.1	*rbsC*	Ribose ABC transporter permease	AMA54098.1	*rbsD*	d-Ribose pyranase	AMA54096.1	*rpiB*	Ribose 5-phosphate isomerase B	AMA54781.1	Glucose/Galactose metabolism		Glucose-1-dehydrogenase	AMA51111.1	*glgD*	Glucose-1-phosphate adenylyltransferase	AMA54756.1	*glgD*	Glucose-1-phosphate adenylyltransferase	AMA53605.1	*galU*	UTP-glucose-1-phosphate uridylyltransferase	AMA52506.1	*galU*	UTP-glucose-1-phosphate uridylyltransferase	AMA54776.1	*G6PD*	Glucose-6-phosphate dehydrogenase	AMA52911.1	*GlcU*	Glucose transporter	AMA51110.1	*G1p*	Glucose-1-phosphate thymidylyltransferase	AMA54296.1		dTDP-glucose 4,6-dehydratase	AMA54295.1	*galE*	UDP-glucose 4-epimerase	AMA54393.1	*PTS_EIIA_1*	PTS glucose transporter subunit IIA	AMA54717.1	*PtsG1*	PTS glucose transporter subunit IICBA	AMA52082.1	*PtsG1*	PTS glucose transporter subunit IICBA	AMA50952.1		Galactose-1-phosphate uridylyltransferase	AMA54324.1	*galT2*	Galactose-1-phosphate uridylyltransferase	AMA54324.1	*GalK*	Galactokinase	AMA54325.1	*CelF*	alpha-Glucosidase/alpha-galactosidase	AMA53548.1	*ugd*	UDP-Glucose 6-dehydrogenase	AMA54054.1	Fructose Metabolism	*glpX*	Fructose 1,6-bisphosphatase	AMA54221.1		Fructose-6-phosphate aldolase	AMA54223.1	*PTS-IIA*	PTS fructose transporter subunit IIA/mannose/fructose/sorbose family	AMA52132.1	*PTS-IIb*	PTS fructose transporter subunit IIB	AMA53179.1		PTS mannose transporter subunit IIABC	AMA51887.1
		*glpX*	Fructose 1,6-bisphosphatase	AMA54221.1		Fructose-6-phosphate aldolase	AMA54223.1	*Fbp2*	Fructose 1,6-bisphosphatase	AMA54529.1	Xylose Metabolism	*xylA*	Xylose isomerase	AMA51266.1	*xylA*	Xylose isomerase	AMA52439.1	l-Arabinose Metabolism	*UgpE*	Arabinose transporter permease	AMA53401.1	*UgpA*	Arabinose transporter permease	AMA53402.1	*UgpB*	Arabinose-binding protein	AMA53403.1	*AraA*	l-Arabinose isomerase	AMA53408.1	*AraJ*	Arabinose ABC transporter permease	AMA51217.1	Mannose Metabolism	*manA*	Mannose-6-phosphate isomerase	AMA51295.1		Mannose-6-phosphate isomerase	AMA51888.1		Mannose-6-phosphate isomerase	AMA54082.1		PTS mannose transporter subunit IID	AMA53177.1	*PTS-IIC*	PTS mannose fructose n-acetylgalactosamine-transporter subunit IIC	AMA53178.1		PTS mannose/fructose/sorbose transporter subunit IIC	AMA53178.1	Rhamnose Metabolisim	*RhaA*	l-Rhamnose isomerase	AMA53625.1		l-Rhamnose isomerase	AMA53625.1	*RhaM*	l-Rhamnose mutarotase	AMA53626.1
Di- and oligosaccharides	Trehalose Metabolism	*PTS_EIIC*	Trehalose permease IIC protein	AMA51474.1	*treC*	Trehalose-6-phosphate hydrolase	AMA51475.1	Lactose/galactose oligosaccharides Metabolism	*galA*	alpha-Galactosidase	AMA53548.1	Maltose Metabolism	*malG*	Maltose ABC transporter permease	AMA53547.1		Maltose phosphorylase	AMA53959.1	Cellobiose Metabolism		PTS system, lactose/cellobiose specific IIA subunit;	AMA54365.1		*celA*	Phosphotransferase system cellobiose-specific component IIB	AMA54366.1			Phosphotransferase system cellobiose-specific component IIB	AMA54367.1		*PtsG1*	PTS system, sucrose-specific IIBC component	AMA54311.1	Sucrose Metabolism		PTS system, sucrose-specific IIBC component	AMA54346.1		*SacC*	Sucrose-6-phosphate hydrolase	AMA54310.1		*sacT*	sac operon transcriptional antiterminator SacT	AMA54313.1
Organic acids	Lactate Metabolism	*ldh*	l-Lactate dehydrogenase	AMA51019.1	*lutP*	l-Lactate permease/uptake of l-lactate	AMA53925.1	*lctP*	l-Lactate permease	AMA51020.1	*LutC*	l-Lactate utilization protein LutC	AMA53909.1
Sugar alcohols	Mannitol Metabolism		PTS mannitol transporter subunit IIBC	AMA51115.1		PTS mannitol transporter subunit IIA	AMA51116.1	*mtlD*	Mannitol-1-phosphate 5-dehydrogenase	AMA51117.1	Inositol Metabolism		Inositol monophosphatase	AMA52157.1	*idh*	Inositol 2-dehydrogenase	AMA53252.1		Inositol 2-dehydrogenase	AMA54485.1	Glycerol Metabolism	*glpT*	Glycerol-3-phosphate transporter	AMA50933.1	*glpP*	Glycerol uptake operon antiterminator GlpP	AMA51623.1	*glpK*	Glycerol kinase GlpK	AMA51625.1	*glpA*	Glycerol-3-phosphate dehydrogenase	AMA51626.1		Acyl-phosphate glycerol 3-phosphate acyltransferase	AMA51650.1	*gpsA*	Glycerol-3-phosphate dehydrogenase	AMA52797.1	*G1pdh*	Glycerol-1-phosphate dehydrogenase	AMA53404.1	*ugpC*	sn-Glycerol-3-phosphate ABC transporter ATP-binding protein	AMA53763.1
Aminosugars	Chitin and N-acetylglucosamine Metabolism	*nagA*	N-Acetylglucosamine-6-phosphate deacetylase	AMA53999.1	*murB*	UDP-N-acetylmuramate dehydrogenase	AMA52215.1		UDP-N-acetylglucosamine 1-carboxyvinyltransferase	AMA54188.1		UDP-N-acetylglucosamine 1-carboxyvinyltransferase	AMA54222.1	*wecB*	UDP-N-acetyl glucosamine 2-epimerase	AMA54062.1		UDP-N-acetyl glucosamine 2-epimerase	AMA54062.1		GlmU, N-acetylglucosamine-1-phosphate-uridyltransferase	AMA50776.1		Glucosamine-6-phosphate deaminase	AMA50953.1
Polysaccharides	Starch Metabolism	*amyA*	alpha-Amylase	AMA51018.1
*Protein metabolism*				
Proteases		*clpA*	ATP-dependent Clp protease ATP-binding subunit	AMA52061.1
		*clpP*	Clp protease	AMA53956.1	*clpC*	ATP-dependent Clp protease	AMA50811.1	Cysteine protease	*yraA*	Cysteine protease	AMA53175.1	Peptidase-S8	*prtP*	Cell wall bound proteinase	AMA52010.1	AMA54710.1	AMA54315.1	AMA52222.1	AMA51767.1	Peptidase-S9	*prtC*	Collagenase-like protease	AMA53208.1	AMA53209.1	*AprE*	Subtilisin	AMA51724.1	*PrsW*	Glutamic-type intramembrane protease	AMA52808.1	Serine protease		Serine protease	AMA51981.1	*htrC*	AMA54548.1	*htrB*	AMA53806.1	*aprX*	AMA52401.1	Metallopeptidases	*htpX*	Zinc metalloprotease	AMA52039.1		Extracellular metalloprotease	AMA50941.1	Family M16		Insulinase	AMA52361.1	AMA52374.1	AMA52373.1	AMA54254.1
Peptide metabolism	Oligopeptide transporters	*oppA*	Oligopeptide ABC transporter	AMA51833.1	*oppB*	Oligopeptide ABC transporter	AMA51834.1	*oppD*	Oligopeptide ABC transporter	AMA51836.1	*oppF*	Oligopeptide ABC transporter	AMA51837.1	Dipeptide transporters	*dppA*	d-aminopeptidase	AMA51983.1	*dppB*	Peptide ABC transporter permease	AMA51984.1	*dppC*	Dipeptide ABC transporter permease	AMA51985.1	*dppD*	Peptide ABC transporter ATP-binding protein	AMA51826.1	AMA51986.1	*dppF*	ATP-binding protein	AMA51827.1	*dppE*	ABC transporter permease	AMA51987.1	Serine peptidase	*Peptidase S9*	Prolyl oligopeptidase	AMA52159.1	AMA53576.1	AMA53731.1	Aminopeptidases	*pcp*	Pyrrolidone-carboxylate peptidase	AMA50980.1		Aminopeptidase	AMA52136.1	*pepA*	Glutamyl aminopeptidase	AMA53409.1			AMA53507.1
		*Map*	Methionine peptidase	AMA51468.1			AMA50862.1	Metallopeptidases (M29-superfamily)	*pepB*	Leucyl aminopeptidase	AMA53713.1	Proline Peptidase	*pepP*	XAA-pro peptidase	AMA52078.1			AMA52970.1	Endopeptidase	*pepF*	Oligoendopeptidase F	AMA51844.1			AMA53803.1	Dipeptidase	*pepV*	Dipeptidase PepV	AMA53518.1	Tripeptidase	*pepT*	Peptidase T	AMA54397.1	Carboxypeptidase	*LdcA*	ld-carboxypeptidase	AMA51988.1	AMA52622.1	AMA52665.1		M32 peptidase; carboxypeptidase	AMA52727.1
*Lipid metabolism*					Lipases and Esterases					Esterase	*EstA*	Triacylglycerol esterase/lipase EstA	AMA51528.1	AMA50985.1	*GDPD*	Glycerophosphodiester	AMA50932.1	AMA51658.1	*YaeI*	Phosphodiesterase	AMA52028.1	AMA52100.1		Carboxylesterase	AMA50943.1	AMA52658.1	AMA53875.1	AMA53942.1		GDSL family lipase	AMA54420.1		Patatin	AMA52194.1	Lipase		Lipase	AMA54515.1	*pldB*	Phospholipase	AMA53569.1

All data presented is generated from this study.

#### 3.2.3. *In silico* analysis of DE111^®^ suggests vitamin and amino acid biosynthetic potential

Bacteria as a supplier of exogenous amino acids and vitamins is becoming a focus point to obtain novel fermented foods with enhanced nutrition availability to the host and the gut microbiota (Preidis et al., 2011). Genome analysis of DE111^®^ has revealed the presence of *de novo* synthesis of water and fat-soluble vitamins and amino acids including five essential amino acids. Genes encoding proteins associated with the synthesis of B-vitamins like B1 (Thiamine), B2 (Riboflavin), B6 (Pyridoxin), B7 (Biotin), B9 (Folate), and vitamin K2 (Menaquinone) were detected in DE111^®^ genome (Table 4). Furthermore, DE111^®^ harbors genes for synthesis of five essential amino acids (threonine, tryptophan, methionine, leucine, and lysine) and two amino acids (cysteine and arginine; Table 4). These findings suggest that DE111^®^ could be considered as a potential strain for preparation of functional foods, offering antioxidant potential and synthesis of vitamins and amino acids.

**Table 4:** Vitamins and amino acid biosynthesis genes detected in DE111^®^ genome.

*De novo* synthesis	Vitamin/amino acid	Product	Protein accession number
Vitamin biosynthesis	Thiamine	Thiamine pyrophosphate-binding protein	AMA51149.1	Thiamine monophosphate kinase *thiL*	AMA51298.1	Phosphomethylpyrimidine synthase *thiC*	AMA51579.1	Thiamine biosynthesis protein *thiS*	AMA51859.1	Thiamine permease	AMA52012.1	Thiamine biosynthesis protein *thiI*	AMA53481.1	Energy-coupled thiamine transporter *thiT*	AMA53607.1	Thiamine-phosphate diphosphorylase *thiE*	AMA54335.1	Hydroxymethylpyrimidine/phosphomethylpyrimidine kinase *thiD*	AMA51862.1	Thiamine pyrophosphokinase *thiN*	AMA54697.1	Thiazole synthase *thiG*	AMA51860.1	Riboflavin	3,4-Dihydroxy-2-butanone 4-phosphate synthase ribB	AMA52838.1	6,7-Diimethyl-8-ribityllumazine synthase *ribE*	AMA52836.1	ATP phosphoribosyltransferase	AMA53990.1	Diaminohydroxyphosphoribosylaminopyrimidine deaminase/5-amino-6-(5-phosphoribosylamino) uracil reductase	AMA52840.1	NADH dehydrogenase ribD	AMA53718.1	Orotidine 5′-phosphate decarboxylase	AMA52246.1	Phosphoribosyl-AMP cyclohydrolase/Phosphoribosyl-ATP pyrophosphatase	AMA53984.1	Riboflavin kinase/FMN adenylyltransferase ribC	AMA52357.1	Riboflavin transporter FmnP	AMA52819.1	Riboflavin synthase subunit alpha	AMA52839.1	Ribulose-phosphate 3-epimerase	AMA53406.1	tRNA pseudouridine synthase B	AMA52356.1	Pyridoxin	D-3-Phosphoglycerate dehydrogenase	AMA52820.1	Pyridoxal 5′-phosphate synthase subunit PdxS	AMA50736.1	1-Deoxy-d-xylulose 5-phosphate synthase	AMA52952.1	Pyridoxal 5′-phosphate synthase glutaminase subunit PdxT	AMA50737.1	Biotin	Acetoacetyl-CoA synthetase/long-chain-fatty-acid–CoA ligase	AMA53380.1	Adenosylmethionine-8-amino-7-oxononanoate aminotransferase *bioA*	AMA53540.1	Biotin synthase *bioB*	AMA53537.1	Long-chain-fatty-acid–CoA ligase	AMA51721.1	Biotin carboxyl carrier protein of acetyl-CoA carboxylase	AMA52512.1	Biotin biosynthesis protein *bioY*	AMA51732.1	6-Carboxyhexanoate–CoA ligase *bioW*	AMA53541.1	8-Amino-7-oxononanoate synthase *bioF*	AMA52387.1	Dethiobiotin synthase *bioD*	AMA53538.1	Folate	Dihydrofolate synthase/folylpolyglutamate synthase folC	AMA53331.1
		GTP cyclohydrolase I type 1	AMA51047.1	Pantoate—beta-alanine ligase	AMA52757.1	Aspartate 1-decarboxylase	AMA52756.1	2-Amino-4-hydroxy-6-hydroxymethyldihydropteridine pyrophosphokinase folK	AMA50804.1	Dihydroneopterin aldolase folB	AMA53858.1	Cell division protein FtsH	AMA50794.1	Hypoxanthine-guanine phosphoribosyltransferase	AMA54632.1	tRNA(Ile)-lysidine synthetase	AMA50793.1	Menaquinone	Menaquinone-specific isochorismate synthase MenF	AMA53703.1	Isochorismate synthase	AMA53707.1	2-Succinyl-6-hydroxy-2,4-cyclohexadiene-1-carboxylate synthase MenD	AMA53598.1	o-Succinylbenzoate synthase MenC	AMA53595.1	o-Succinylbenzoyl-CoA MenE	AMA53596.1	1,4-Dihydroxy-2-naphthoyl-CoA MenB	AMA53597.1	1,4-Dihydroxy-2-naphthoate octaprenyltransferase MenA	AMA54356.1	Demethylmenaquinone methyltransferase MenG	AMA52789.1
Essential amino acid biosynthesis	Threonine	Threonine–tRNA ligase	AMA54267.1	Homoserine dehydrogenase	AMA53733.1	Aspartate aminotransferase	AMA54281.1	Aspartate-semialdehyde dehydrogenase	AMA52365.1	Homoserine kinase	AMA53732.1	Threonine synthase	AMA54760.1	Tryptophan	Tryptophan synthase subunit alpha	AMA52778.1	Tryptophan synthase subunit beta	AMA52779.1	Phosphoribosylformimino-5-aminoimidazole carboxamide ribotide isomerase	AMA53986.1	Isochorismatase	AMA53104.1	Indole-3-glycerol phosphate synthase	AMA52781.1	Anthranilate phosphoribosyltransferase	AMA52782.1	Anthranilate synthase, aminase component	AMA52783.1	Methionine	Cysteine synthase	AMA53200.1	Cystathionine beta-lyase	AMA53655.1	Cystathionine gamma-synthase	AMA54677.1	S-Adenosylmethionine synthetase	AMA53573.1	Serine acetyltransferase	AMA50818.1	Homoserine O-succinyltransferase	AMA52710.1	Methionine ABC transporter ATP-binding protein	AMA53781.1	Homoserine kinase	AMA53732.1
		Homoserine dehydrogenase	AMA53733.1	5-Methyltetrahydropteroyltriglutamate—homocysteine methyltransferase	AMA54401.1	5-Methyltetrahydrofolate—homocysteine methyltransferase	AMA51788.1	5,10-Methylenetetrahydrofolate reductase	AMA52956.1	Leucine	3-Isopropylmalate dehydrogenase	AMA53351.1	3-Isopropylmalate dehydratase small subunit	AMA53350.1	3-Isopropylmalate dehydratase large subunit	AMA53350.1	2-Isopropylmalate synthase	AMA53352.1	Lysine	Aspartokinase		4-Hydroxy-tetrahydrodipicolinate synthase	AMA52367.1	4-Hydroxy-tetrahydrodipicolinate reductase	AMA52764.1	Aspartate-semialdehyde dehydrogenase	AMA52365.1	Diaminopimelate decarboxylase	AMA54724.1	N-Acetyl-L,L-diaminopimelate deacetylase	AMA52112.1	2,3,4,5-Tetrahydropyridine-2,6-dicarboxylate N-acetyltransferase	AMA52111.1	N-Acetyl-L,L-diaminopimelate aminotransferase	AMA52050.1	Aspartate-semialdehyde dehydrogenase	AMA52365.1
Other amino acids	Cysteine	Cysteine synthase A	AMA50798.1	Cystathionine beta-lyase	AMA53655.1	Cystathionine gamma-synthase	AMA54677.1	Serine acetyltransferase	AMA50818.1	Sulfite reductase [NADPH] hemoprotein beta-component	AMA53853.1	Sulfite reductase [NADPH] flavoprotein alpha-component	AMA53854.1	Phosphoribosyl-AMP cyclohydrolase	AMA53984.1	Imidazole glycerol phosphate synthase cyclase subunit	AMA53985.1	Phosphoribosylformimino-5-aminoimidazole carboxamide ribotide isomerase	AMA53986.1	Imidazole glycerol phosphate synthase subunit HisH	AMA53987.1	Imidazoleglycerol-phosphate dehydratase	AMA53988.1	ATP phosphoribosyltransferase	AMA53990.1	Histidinol-phosphate aminotransferase	AMA52777.1	Arginine	N-Acetyl-gamma-glutamyl-phosphate reductase	AMA51809.1	N-Acetylglutamate synthase	AMA51810.1	Acetylglutamate kinase	AMA51811.1	Acetylornithine aminotransferase	AMA51812.1	Ornithine carbamoyltransferase	AMA51815.1	Argininosuccinate synthase	AMA53469.1	Argininosuccinate lyase	AMA53468.1	Arginine pathway regulatory protein ArgR	AMA52950.1

All data presented is generated from this study.

#### 3.2.4. Bacteriocins and other competitive exclusion mechanisms present in DE111^®^

The gastrointestinal tract harbors a vast array of competing microbes and the ability of DE111^®^ to produce antimicrobial peptides, would be advantageous for its survival in the gut by competitive exclusion. To identify bioactive metabolites in DE111^®^, the AntiSMASH tool was employed. This prediction tool has been used in previous studies for the evaluation of known, distinct and new bioactive metabolites in *B. subtilis* (Medema et al., 2011). A total of 13 biosynthetic gene clusters (BGC) were predicted, of which 8 were encoding antifungal and antimicrobial peptides (Table 5). The five antibacterial clusters identified were Sublacin, Subtilosin A, Lactococcin, Bacilysin and Bacillibactin. Additionally, a cluster encoding bacterial cyclic lipopeptide, surfactants and two clusters encoding two antifungal cyclic lipopeptides (bacillomycin F and fengycin) were also identified. These data correlate with findings of a previous study, using comparative genomic and metabolomic approaches to determine the production of antimicrobial and antifungal peptides by two *B. subtilis* strains, one of which was DE111^®^ (Knight et al., 2018).

**Table 5:** List of biosynthetic clusters of secondary metabolites identified in *Bacillus subtilis* DE111^®^ genome.

Compound	Predicted function	Core protein GenBank accession number
Surfactin	Multiple	AMA51066.1
		AMA51067.1
Fengycin	Antifungal	AMA52521.1
Bacillomycin	Antifungal	AMA52501.1
		AMA52503.1
		AMA52504.1
Sublacin	Antibacterial	AMA52595.1
Bacillibactin	Siderphore	AMA53705.1
Subtilosin A	Antibacterial	AMA54248.1
		AMA54249.1
Lactococcin	Antibacterial	AMA52608.1
Bacilysin	Antibacterial	AMA54280.1

### 3.3. *In vitro* screening for potential probiotic activities of DE111^®^

Herein, the results from the *in vitro* analysis of DE111^®^ to survive harsh conditions, adhere to intestinal cell, enzymatic profile, antioxidant, and antimicrobial activities are discussed.

#### 3.3.1. DE111^®^ spores displays strong ability to survive harsh temperatures

**Figure 2:**
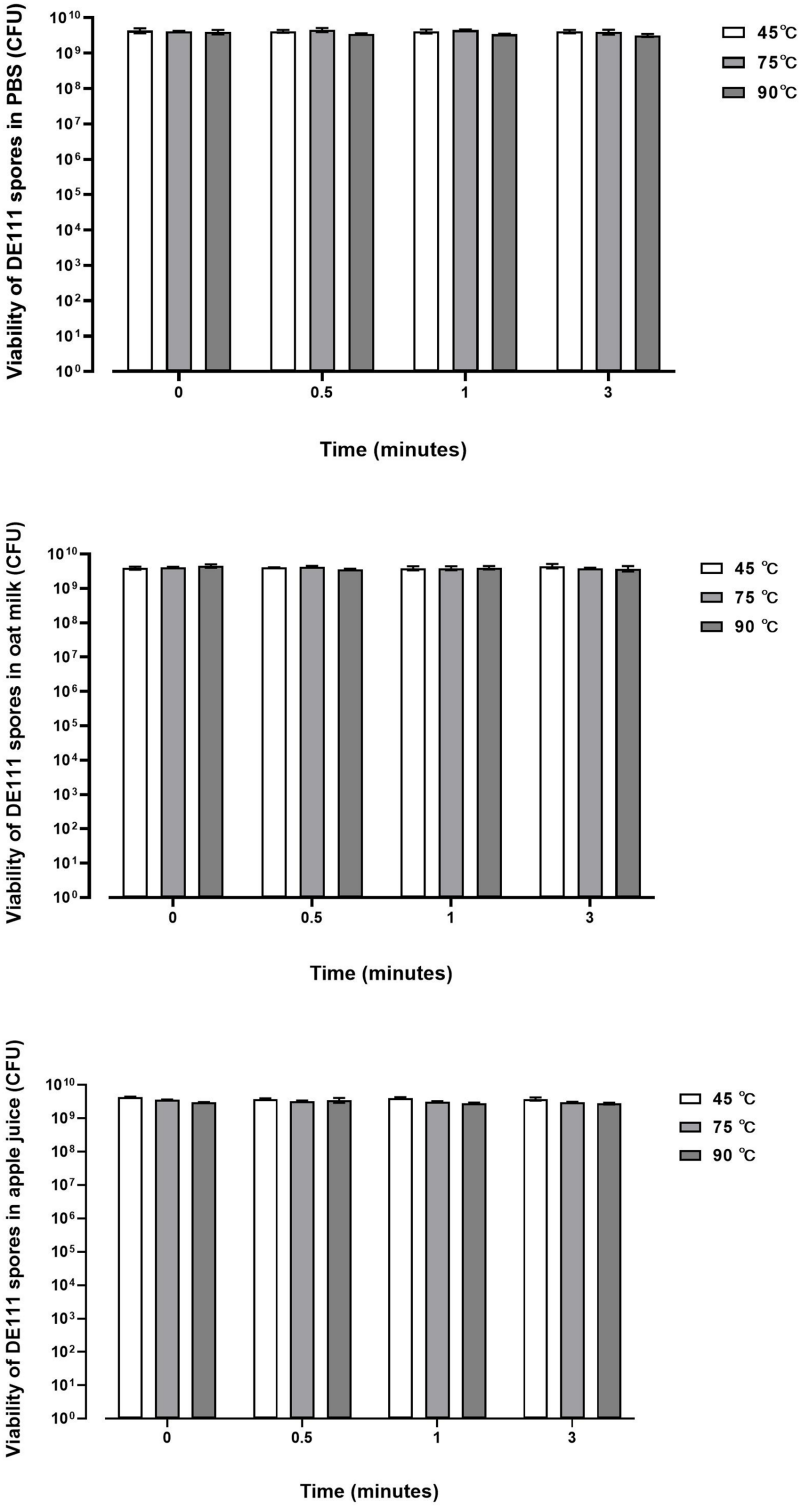
DE111^®^ is resistant to temperature challenges in PBS, Oat milk and Apple juice during pasteurization process. Results show average concentration (*N* = 3) ± standard error.

The ability of DE111 to withstand different pasteurization temperatures (45, 75, and 90°C) in 1× PBS, Oat milk and 5% Apple juice was investigated at 4-time points: 0, 0.5, 1, and 3 min. The viability of DE111^®^ in 1× PBS and oat milk remained stable at 45, 75, and 90°C at all time points (Figure 2). The viability of DE111^®^ in 5% apple juice at pH 4.18 also remained stable at 45, 75, and 90°C at all time points (Figure 2). These results confirm that DE111^®^ spores can survive pasteurization process at 45, 75, and 90°C.

#### 3.3.2. DE111^®^ adhere to mucous secreting intestinal epithelial cell line

**Figure 3:**
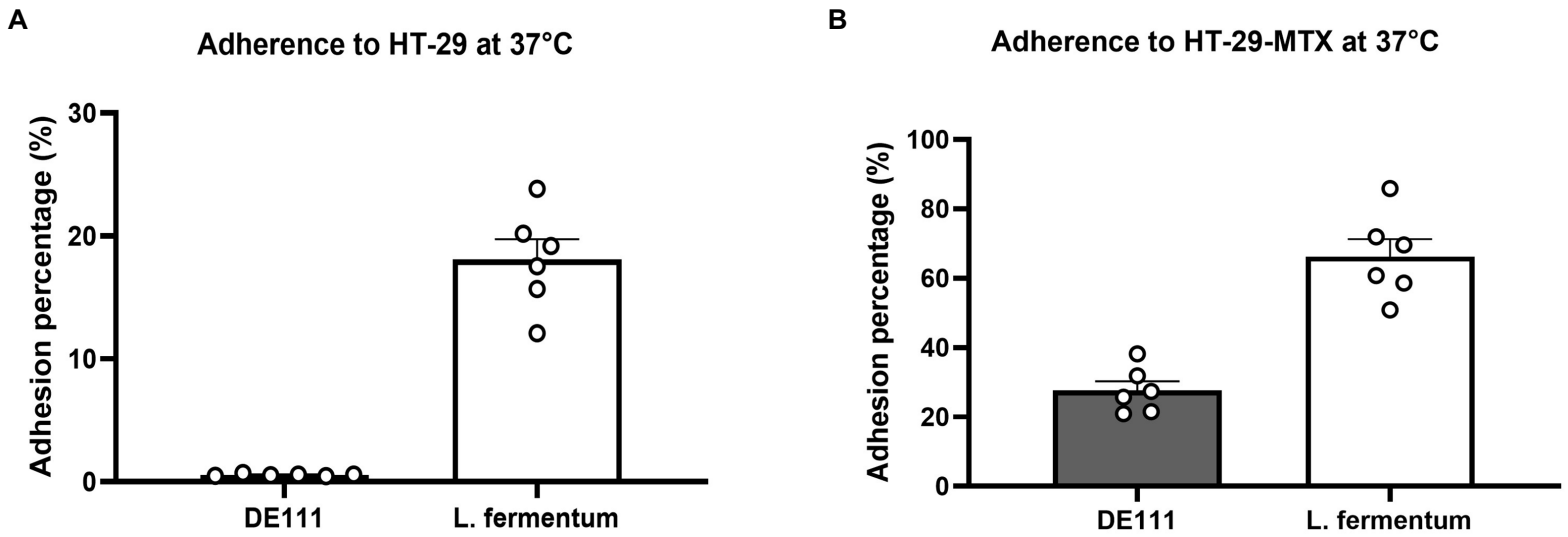
**(A)** Adherence of DE111^®^ and *Lactobacillus fermentum* (control) to HT-29 cells at 37°C, and **(B)** adherence of DE111^®^ and *L. fermentum* (control) to HT-29 cells at 37°C. (*N* = 6) ± standard error.

To determine whether DE111^®^ exhibited adhesive ability, corroborating the *in-silico* data, microbial adhesion to human colon adenocarcinoma cell line HT-29 and mucus-secreting HT29-MTX cell model was performed. These cell lines are widely used for the evaluation of adhesion properties of probiotic strains (Gagnon et al., 2013). *Lactobacillus fermentum* was used as a positive control as its ability to adhere to intestinal cell lines is well established (Archer et al., 2018). The adhesion of DE111^®^ to HT29-MTX cell model was significantly higher that the HT-29 cells at 37°C, likely due to high adhesiveness of this strains to mucins present in the native human mucus layer covering the whole cell surface, compared to the low-mucus producing HT-29 cell models (Figure 3).

#### 3.3.3. High proteolytic action of DE111^®^ releasing diverse range of amino acids from milk protein hydrolysis

**Figure 4:**
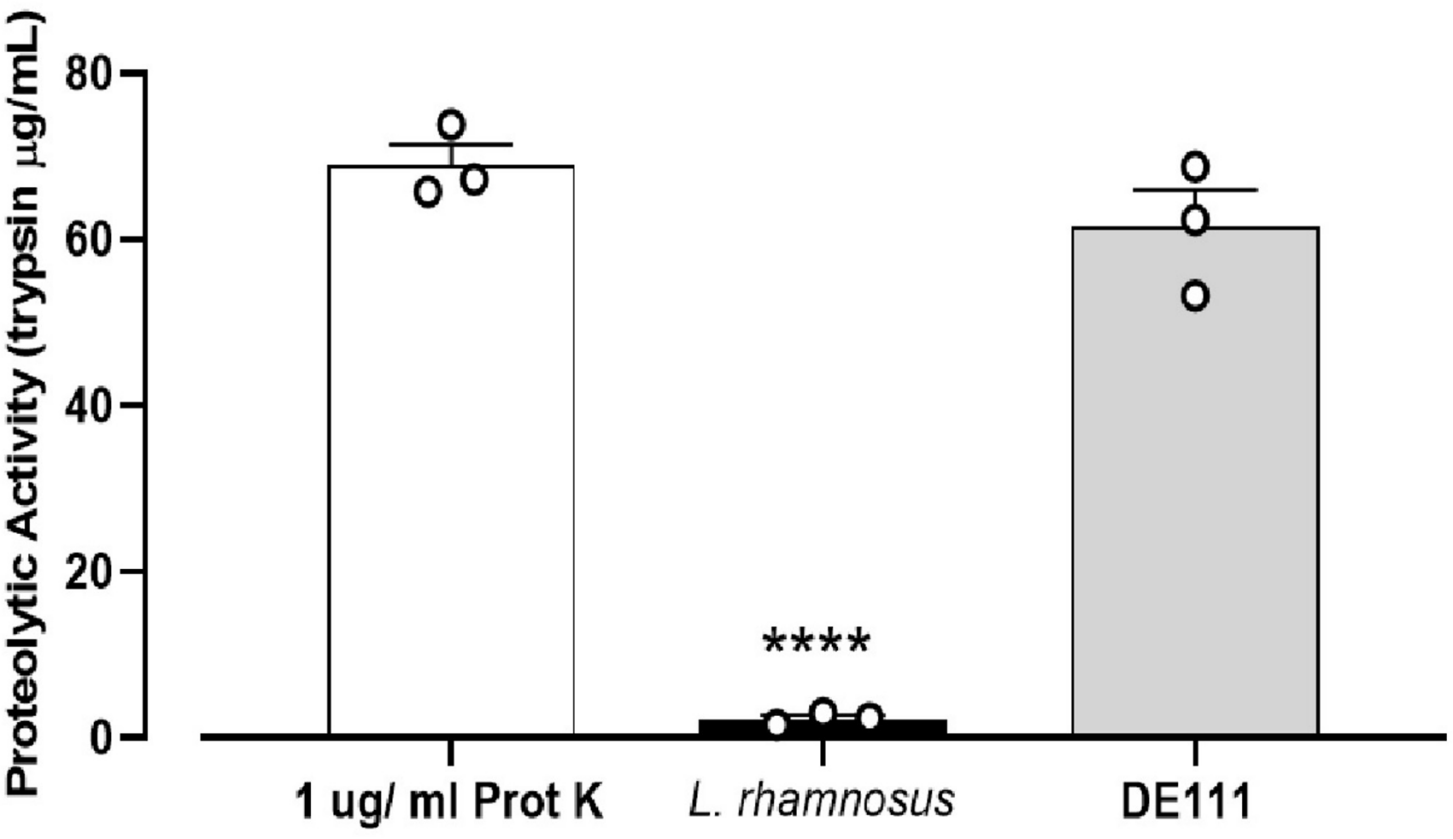
High proteolytic activity of DE111^®^ determined by EnzCheck^®^ kit following incubation at 37°C for 24 h. Experiments were performed in three biological replicates with three technical replicates. The graph presents average activities (*N* = 3 ± SEM) of DE111^®^, *Lactobacillus rhamnosus* strains, and Proteinase K (positive control). Symbol; ****p < 0.0001.

*In vitro* screening of the DE111^®^ proteolytic activity toward casein degradation using skim milk agar plates was performed and analyzed using a kit based on the proteolysis of fluorescently labeled casein derivatives. Casein is a rich source of essential amino acids, and its breakdown serves a vital role in human physiology (Mohanty et al., 2016). Preliminary analysis of casein degradation was conducted using skim milk agar plates. Development of extensive zones of clearing were observed around the growth of DE111^®^ and no clearing were seen with the comparator probiotic strain of *L. rhamnosus* GG after 24 h incubation (Supplementary Figure S1). To quantify the level of this protease activity demonstrated by DE111^®^, a kit based on the proteolysis of BODIPY^®^ FL-labeled casein derivatives was employed. DE111^®^ demonstrated superior protease activity to *L. rhamnosus* when measured by this assay, the levels varied significantly between the two strains (Figure 4). The concentration of trypsin equivalent protease activity ranged from 61.5 μg/ml for DE111^®^ to 2.3 μg/ml for *L. rhamnosus*.

**Figure 5:**
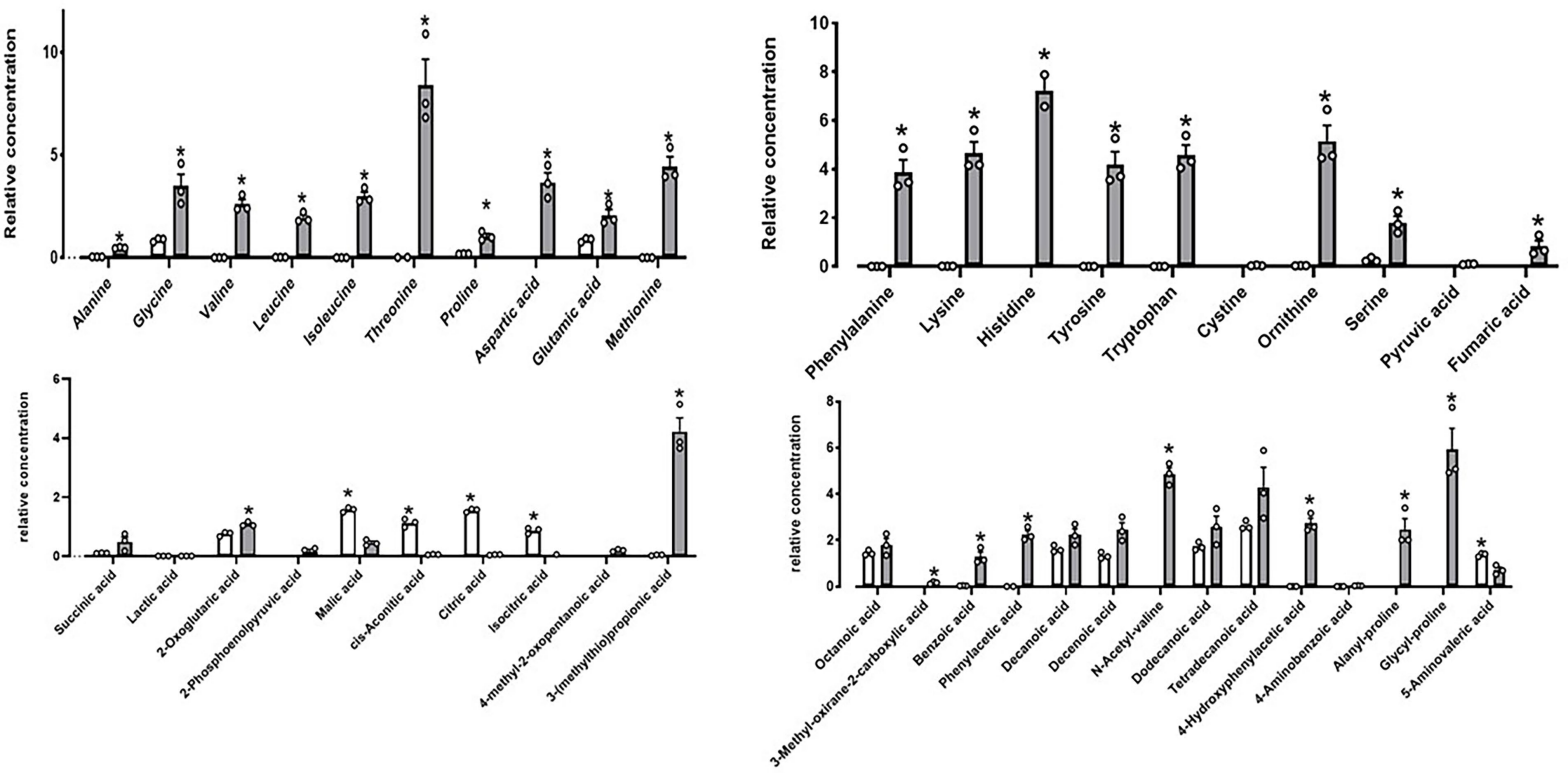
DE111^®^ increased free amino acid concentrations in UHT fermented milk samples. Statistical analysis performed using Multiple T test – using unpaired parametric, Two-stage step-up (Benjamini, Krieger, and Yekutieli) and *p-value ≤0.01. The graph presents average activities (*n* = 3 ± SEM). The white bar represents the control and gray bar represent DE111^®^.

Furthermore, metabolites detected after DE111^®^ UHT milk fermentation presented strong association with the extensive protease activity. A total of 44 compounds were identified, out of which 32 were significantly altered by DE111^®^ (Figure 5). The majority of the compounds identified were associated with amino acid catabolism pathways (Tavaria et al., 2002). These include a few of the aromatic carboxylic acids such as 4-methyl-2-oxopentanoic acid, 3-(methylthio) propionic acid and benzoic acid, which are a result of further downstream catabolism of free amino acids such as leucine, alanine and phenylalanine (Ardö, 2006). The potential precursors or the peptidases that could lead to the release of these amino acids/compounds are identified and presented in Supplementary Table S3. In addition, the protease action of DE111^®^ in UHT milk caused a strong coagulation after 48 h incubation at pH values (5.8–5.9) well above those defined for acid-induced coagulation (pH ~4.6; Gigante et al., 2006). This further highlights the extensive milk protein hydrolysis capability of DE111^®^. Altogether, the high casein degrading ability, the range of proteases, the peptide transporters and peptidases identified in DE111^®^, and the metabolites produced from the fermentation of UHT milk suggested that this organism has a strong proteolytic system.

#### 3.3.4. Enzymatic and metabolomic analysis of DE111^®^ indicates esterolytic and oligosaccharide degradation capability

**Figure 6:**
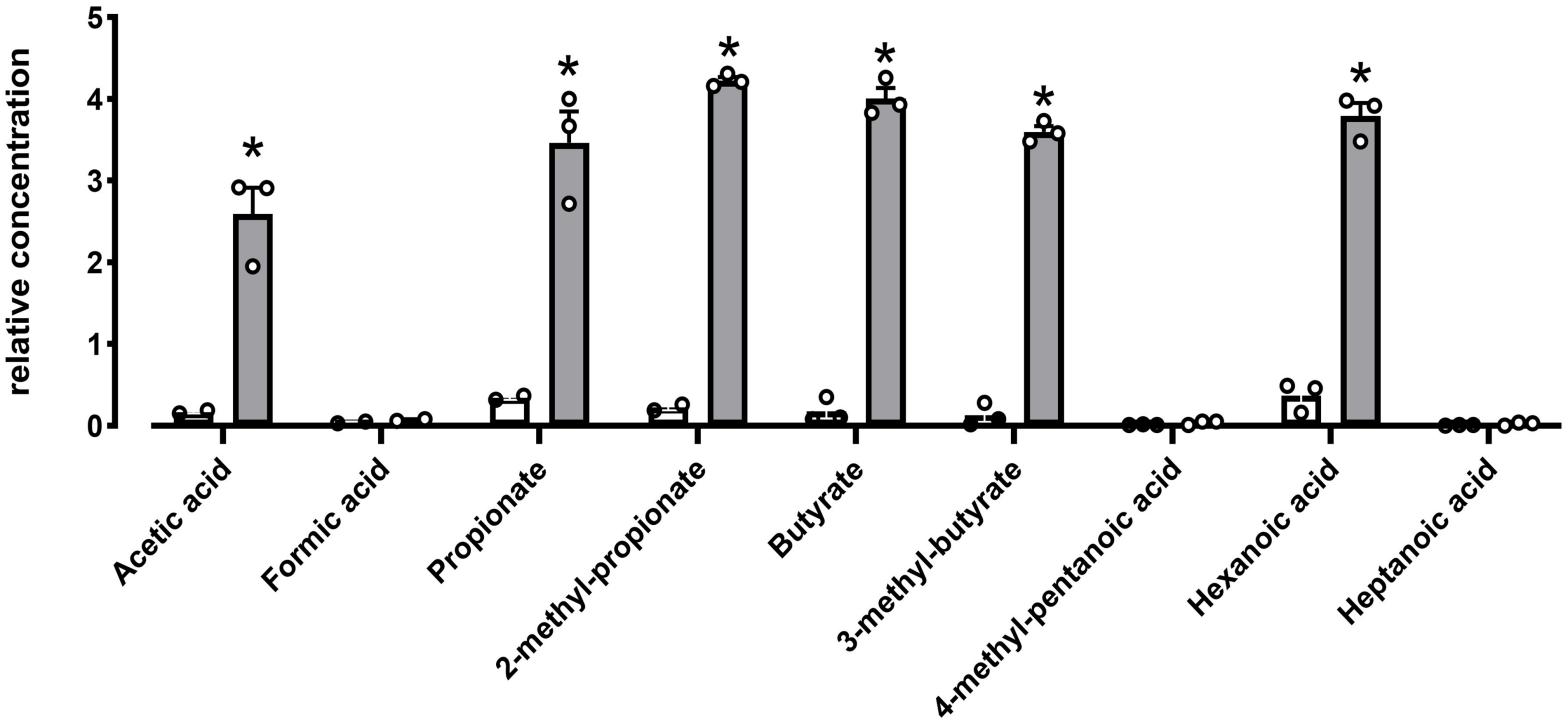
SCFA increased in DE111^®^ UHT fermented milk samples. Statistical analysis performed using Multiple T test – using unpaired parametric, Two-stage step-up (Benjamini, Krieger, and Yekutieli) and *p-value ≤0.01. The graph presents average activities (*N* = 3 ± SEM). The white bar represents the control and gray bar represent DE111^®^.

Hydrolytic activities toward lipases, esterase’s and carbohydrate were evaluated using the semi-quantitative API-ZYM and API 50 CH kit system. DE111^®^ was positive for 18 carbohydrates out of 49 tested, which includes Glycerol, d-Ribose, l-Arabinose, d-Xylose, d-Glucose, d-Fructose, d-Mannose, Inositol, d-Mannitol, d-Maltose, d-Saccharose, d-Sorbitol, d-Cellobiose, Sucrose, d-Trehalose, Amygdalin, Arbutin, Salicin, and Esculin Ferric Citrate (Supplementary Table S4). Investigation using API-ZYM kit system indicated DE111^®^ was positive for esterase (C4 and C8), phosphohydrolase, acid and alkaline phosphatase, and galactosidase activity (Supplementary Table S5). Metabolomic analysis of DE111^®^ fermented UHT milk, identified a total of nine short chain fatty acid (SCFA) compounds, of which only six were found to be statistically significant increased by DE111^®^ (Figure 6). The two esters (3-methyl butyrate and 2-methyl propanioate) detected could potentially be synthesized by the esterases and lipases identified DE111^®^ genome. Overall, the metabolomic analysis of DE111^®^ indicates that this strain has lipolytic ability and can lead to generation of esters (reaction of FFA with alcohol).

#### 3.3.5. *In vitro* analysis reveal strong antioxidant capacity of DE111^®^

**Figure 7:**
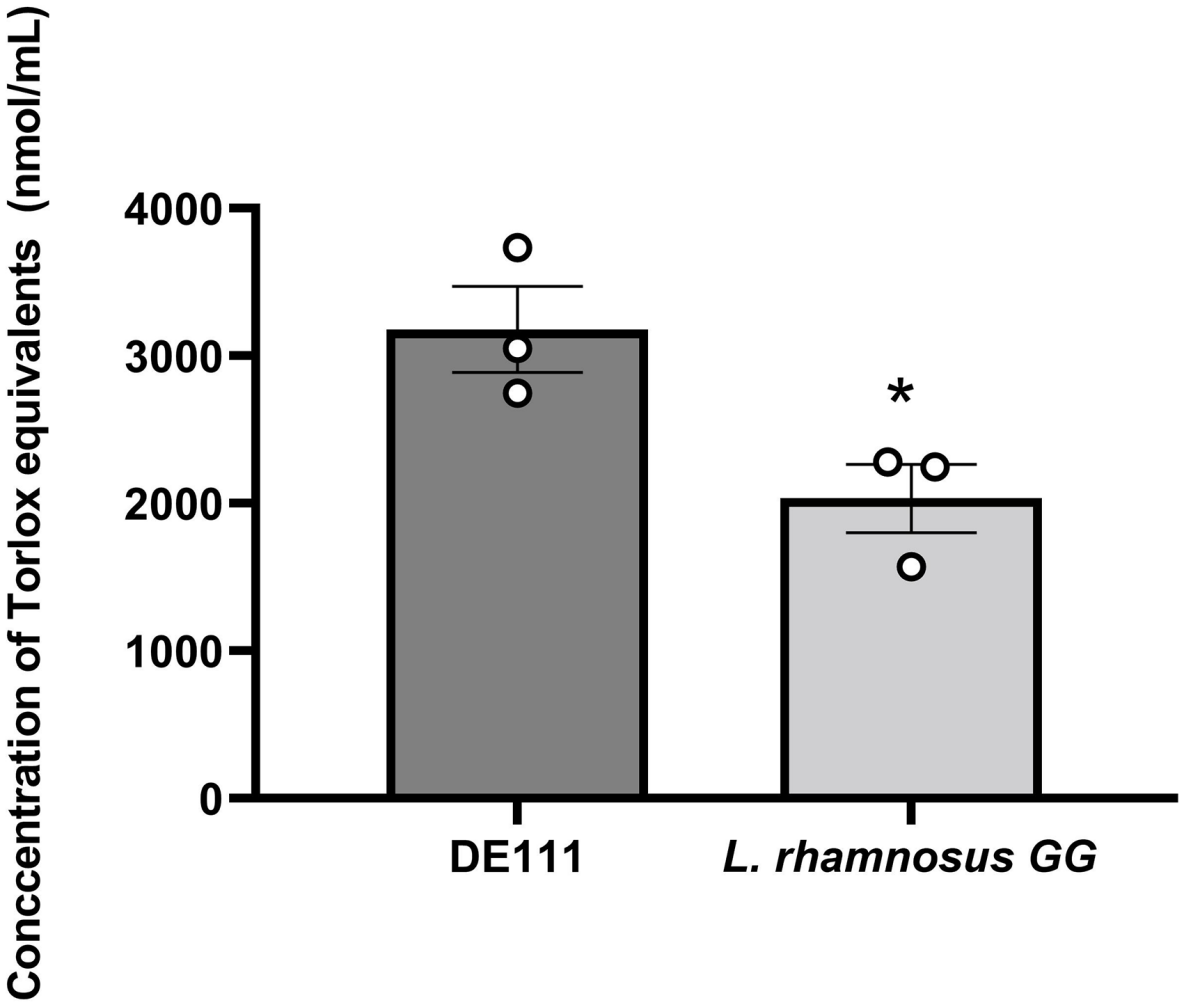
High total antioxidant activity of DE111^®^ in comparison to *Lactobacillus rhamnosus* GG. Statistical analysis performed using unpaired T test and *p-value 0.037. Results show average concentration of Trolox equivalents in nmole/ml (*n* = 3) ± standard error.

Oxidants, such as reactive oxygen species (ROS) and reactive nitrogen species (RNS), can generate free radicals that can cause severe oxidative damage to cellular lipids, membranes, proteins, and DNA. Antioxidants can scavenge these free radicals and prevent cellular oxidative stress by enzymatic and non-enzymatic mechanisms (Valko et al., 2007). In this study, a total antioxidant capacity assay to characterize DE111^®^ activity was used. In this assay *L. rhamnosus* GG was used as a comparator strain as its antioxidant capacity has been previously reported (Ma et al., 2022). While, both DE111^®^ and *L. rhamnosus* GG demonstrated antioxidant activity when measured by this assay, the levels varied significantly between the two strains (Figure 7). The concentration of Trolox equivalent antioxidant activity ranged from 3,178.54 nmol/ml for DE111^®^ to 2,034.04 nmol/ml for *L. rhamnosus* GG.

#### 3.3.6. DE111^®^ presents antagonistic activity against pathogens

A total of 22 known bacterial pathogens were investigated in this study, out of which DE111^®^ exhibited inhibition zones against 18 pathogens including those that are involved in urinary tract, intestinal and common skin infections such as *P. aeruginosa*, *E. coli, G.vaginalis, S. aureus, S. epidermidis*, *S. warneri*, and *C. acnes* (Table 6 and Supplementary Figure S2). Results from this study demonstrated a broad antimicrobial profile of DE111^®^, validating the antagonistic nature of DE111^®^ proved by genomic data.

**Table 6:** *Bacillus subtilis* DE111^®^ antimicrobial activity against urinary tract, intestinal, and common skin pathogens.

Indicator strain	Zone of inhibition
*Pseudomonas aeruginosa* DSM3227	+
*Yersinia enterocolitica* DSM4780	−
*Staphylococcus warneri* DSM20316	+
*Escherichia coli* ATCC25922	+
*Staphylococcus epidermidis* DSM20044	+
*Listeria monocytogenes* DSM20600	−
*Shigella flexnerii* DSM4782	+
*Candida albicans* DSM3454	+
*Salmonella enteritidis* ATCC13076	+
*Staphylococcus pseudintermedius* DSM21284	+
*Staphylococcus saprophyticus* DSM20229	+
*Staphylococcus aureus* DSM1104	+
*Staphylococcus aureus* DSM17091	+
*Corynebacterium flavescens* DSM20296	−
*Acinetobacter baumannii* DSM30007	+
*Enterococcus faecalis* DSM20478	−
*Streptococcus agalactiae* DSM2134	+
*Streptococcus pyogenes* DSM20565	+
*Cutibacterium acne* DSM1897	+
*Campilobacter jejuni* DSM4688	+
*Gardernella vaginalis* DSM4944	+
*Bacillus cereus* DSM31	+

Symbols: +, positive for zone of inhibition; −, negative for zone of inhibition; All data presented is generated from this study.

## 4. Discussion

Survival and transient colonization of the GIT are key factors for probiotic strains to exert a sufficient-host interactions to confer health benefits. Therefore, in this current study a combination of genomic, metabolomic, and enzymatic analysis was interrogated to explore the overall capability of *B. subtilis* DE111^®^ to survive harsh gastric transit in addition to its metabolic activities that could provide health benefits to the host.

Genomic analysis was performed to investigate acid and bile tolerance mechanisms as these constitute a significant hurdle for probiotic microorganisms to survive and retain activity in the GIT. DE111^®^ encodes several acid tolerance mechanisms that have been well studied in lactic acid bacteria (LAB; Table 2; Ruiz et al., 2011). These mechanisms are employed to maintain stable intracellular pH in acidic environments including membrane ATPase (F1–F0–ATPase) and lactate dehydrogenase (LDH), both of which are identified in DE111^®^ (Selle and Klaenhammer, 2013). Additionally, DnaK and Enolases which were previously described to be involved in bile modulation during intestinal colonization were also identified (Oliveira et al., 2017). Further, to survive in the digestive tract, resist the intestinal microbiota, possibly colonize the GIT tract, and express specific functions under conditions that are unfavorable to growth requires the ability to quickly respond to stresses (van de Guchte et al., 2002; Tripathi and Giri, 2014). In *Lactobacillus gasseri* ATCC33323 genes encoding GroEL, GroES, DnaK, DnaJ, and Clp protease chaperones were associated to protect against intracellular aggregation of proteins during stress (Selle and Klaenhammer, 2013). Similar genes encoding stress responses proteins in DE111^®^ were found, adding to the ability of this strain to survive under various stress conditions. Genomic insights for stress, acid, and bile tolerance in DE111^®^ has been confirmed by supporting wet lab experiments presented in DE111^®^ Gras Notice no 831. In this Gras Notice, the survivability of DE111^®^ is investigated in simulated gastric juice at pH 1.2 following 24 h incubation. While for bile salt, survivability was measured by exposing DE111^®^ to 0.15, 0.30 and 0.45% (wt/vol) of ox gall supplemented in nutrient broth for a maximum of 24 h. Results from these studies illustrated that the viability of DE111^®^ was not reduced after contact with acidic fluid or acidic/salt concentrated nutrient broth for 24 h. Hence, this study confirms DE111^®^ could tolerate bile salts up to 0.45% and simulated gastric juice of pH 1.2 validating the genomic data. In addition, this previous study has confirmed the ability of orally ingested *B. subtilis* DE111^®^ spores to remain viable during transit through the stomach and germinate in the small intestine of humans within 3 h of ingestion (Colom et al., 2021).

Additionally, the presence of six small acid-soluble spore proteins (SASPs) were identified (Supplementary Table S1). These SAPS produced by *Bacillus* and other *Clostridia* species heave been associated with playing an important role in protecting their spores from heat-damage (Galperin et al., 2012). *In vitro*, the ability of DE111^®^ to withstand different pasteurization temperatures at 45°C, 75°C and 90°C was investigated. As temperatures above 45–50°C during processing are detrimental to probiotic survival (Tripathi and Giri, 2014). Figure 2 shows no reduction in DE111^®^ viability across all three processing temperatures (45, 75, and 90°C) in PBS, Oat milk and Apple juice. Similarly, Majeed et al. (2021), investigated the thermostability of *Bacillus coagulans* MTCC5856 in different media such as buffer, milk, and juice, to correlate with milk pasteurization which is normally practiced at two different temperatures (63°C and 72°C) at different time intervals. The results from this study showed no significant reduction in the viability of *B. coagulans* MTCC5856 in all the three media (milk, buffer, and juice) across the two temperatures for up to 360 min (Majeed et al., 2021). These results correlate with this study, together confirming the thermostability of this strain. Altogether, the genetic ability of DE111^®^, *in vitro* and clinical studies supports the ability of this strain to survive and remain viable under GIT environmental stresses and processing environments.

Adhesion and potential penetration of the mucus layer are important probiotic attributes that may contribute to transient colonization of the GIT and competitive exclusion of pathogens (Selle and Klaenhammer, 2013). Genome analysis of DE111^®^ identified various proteins associated with adhesion and aggregation (Supplementary Table S2). These include chitin binding protein, enolases and γ-PGA which are regarded as common adherence factors found in *Bacillus* species (Marvasi et al., 2010; Qin et al., 2020). To determine whether DE111^®^ exhibited adhesive ability, corroborating the *in-silico* data, microbial adhesion to intestinal epithelial cell lines HT-29 and HT-29-MTX was assessed. Percentage of adherence of DE111^®^ to HT-29-MTX (27.6%) was significantly greater than HT-29 (0.665%). In both, cell lines the adherence of DE111^®^ was lower than that of the reference probiotic strain *L. fermentum*. The results suggests that mucus may play an important role in adhesion of DE111^®^. Nevertheless, DE111^®^ demonstrates moderate activity in adhering to gastrointestinal epithelial cell lining.

It is important for probiotics to have antimicrobial activities to prevent infection by pathogenic bacteria in hosts. A total of 13 biosynthetic gene clusters (BGC) were predicted DE111^®^ genome, of which 8 were encoding antifungal and antimicrobial peptides including five antibacterial clusters; Sublacin, Subtilosin A, Lactococcin, Bacilysin, and Bacillibactin. Sublancin belonging to class of Lantibiotics, have been reported in other studies to be produced by *B. subtilis* strains (Dubois et al., 2009). Sublancin exhibits broad spectrum of bactericidal activity against Gram-positive bacteria, including important pathogens such as *Bacillus cereus*, *Streptococcus pyogenes*, and *Staphylococcus aureus* (Dubois et al., 2009). Bacilysin is a broad-spectrum non-ribosomally produced dipeptide with activity against a wide range of bacteria, some yeasts and also against food borne pathogens (Özcengiz and Öğülür, 2015; Nannan et al., 2021). Subtilosin A and Lactococcin are bacteriocin often reported to be produced by *B. subtilis* and *Lactococcus lactis*, species, which have been shown to be important in ecological niche competition and have antimicrobial activity against *Listeria monocytogenes* (Holo et al., 1991; Shelburne et al., 2007; Hawlena et al., 2012; Kapse et al., 2019). Additionally, a cluster encoding bacterial cyclic lipopeptide, surfactants and two clusters encoding two antifungal cyclic lipopeptides (bacillomycin F and fengycin) were also identified. Surfactins produced by *B. subtlis* probiotic strain PB6 were shown to inhibit phospholipase A2 (PLA2) downregulating pro-inflammatory and upregulating anti-inflammatory cytokines in humans (Selvam et al., 2009). DE111^®^ has been shown to secrete surfactants indicating that this strain may also be able mediate these interactions through lipopeptide secretion. Additionally, DE111^®^ also produced fengycins, an antifungal lipopeptide, that inhibits filamentous fungi but is ineffective against yeast and bacteria (Knight et al., 2018). These are often reported in *Bacillus* spp. and strains isolated as antagonists of plant pathogenic fungi (Vanittanakom et al., 1986). Bacillomycin F was also shown to be secreted by DE111^®^ in the previous study, this is a member of the iturin family of lipopeptides, with antifungal activities, especially against filamentous fungi (Gu et al., 2017; Kapse et al., 2019).

All these genomic findings correlate to previous comparative genomic and metabolomic analysis used to identify DE111^®^ ability to produce antimicrobial and antifungal peptides (Knight et al., 2018). In the current study, antagonistic activity of DE111^®^ to inhibit a total of 18 known skin and enteric opportunistic pathogens was confirmed using the agar overlay assay (Supplementary Figure S2). DE111^®^ exhibited antimicrobial activity against those that are involved in urinary tract, intestinal and common skin infections such as *C. acnes, E. coli, G. vaginalis, P. aeruginosa*, *S. aureus*, *S. epidermidis*, *S. warneri*, *S. flexnerii*, and *S. saprophyticus* (Table 6). Additionally, DE111^®^ displayed antagonistic activity toward *Candida albicans*, an opportunistic pathogenic yeast that is a common member of the human gut microbiota (Table 6). Considering all these findings, DE111^®^ has potential to control the presence of opportunistic pathogens in the gut and urinary tract where semi-liquid to liquid conditions will be common. Moreover, DE111^®^ has the potentially to prevent the spread of opportunistic pathogens on dryer environments like the human skin. Altogether, the results from this study demonstrated a broad antimicrobial profile of DE111^®^, validating the antagonistic nature of DE111^®^ proved by genomic data.

Next, the mechanisms present in DE111^®^ that may play a role in positively impacting on host health were investigated. Dissection of DE111^®^ genome suggests that a relatively large proportion of this genetic arsenal is involved in the metabolism and transport of carbohydrates, proteins, and fats. In this study, DE111^®^ demonstrates the ability to ferment a total of 18 carbohydrates which includes: Glycerol, d-Ribose, l-Arabinose, d-Xylose, d-Glucose, d-Fructose, d-Mannose, Inositol, d-Mannitol, d-Maltose, d-Saccharose, d-Sorbitol, d-Cellobiose, Sucrose, d-Trehalose, Amygdalin, Arbutin, Salicin, and Esculin Ferric Citrate (Supplementary Table S4). Indeed, several transporters and enzymes involved in the metabolism of all except; Amygdalin, Arbutin, Salicin, and Esculin Ferric Citrate (Table 3) were identified. The reasons for this disparity between the genome and *in vitro* analysis could be that (1) there are other binding protein-dependent sugar uptake systems with overlapping substrate specify present in DE111^®^ that could potentially transport these sugars (2) there other enzymes with broad substrate specificity present that could use these sugars such as β-glucosidase can degrade Esculin Hydrate – Ferric ammonium Citrate. On the contrary, there are genes encoding enzymes identified involved in lactate, starch and N-Acetylglucosamine, whereas the current investigation using API 50 CH indicated DE111^®^ could not ferment N-Acetylglucosamine or polysaccharides such as amidon (starch) or Inulin. The reason for this disagreement could be that the set of genes involved in the uptake and metabolism of these sugars are incomplete or in this *in vitro* model the enzymes are not expressed to degrade these carbohydrates. DE111^®^ was also positive for esterase, phosphohydrolase, acid and alkaline phosphatase, and galactosidase activity using API-ZYM strips (Supplementary Table S5). In this study, genes encoding alpha-galactosidase and several esterases, have been identified. Additionally, genes encoding GDSL like lipase is identified, GDSL is defined as an esterase that preferentially hydrolyzes short-chain fatty acids, particularly pNP-acetate (C2) and pNP-butyrate (C4), also weak activity with pNP-hexanoate (C6) pNP-octanoate (C8), and short-chain tryglycerides such as triacetin and tributyrin. This could be the enzyme responsible for the hydrolysis of C4 and C8 substrates by DE111^®^. Alkaline phosphatase and phosphohydrolase are also identified (not reported in the table acc no: AMA50977.1; AMA53088.1, respectively) from the genome. Overall, the presence of several extracellular and hydrolytic enzymes indicates a high potential of DE111^®^ to aid in carbohydrate digestion. Metabolomic analysis of DE111^®^ fermented UHT milk indicated a significant release of short-chain fatty acids (SCFAs) including butyrate and propionate (Figure 6). SCFAs are produced through saccharolytic fermentation of complex resistant carbohydrates such as fructo-oligosaccharides, sugar alcohols, resistant starch, inulin, and polysaccharides from plant cell walls. SCFAs are associated with improving gut barrier integrity, glucose, and lipid metabolism, regulating the immune system, the inflammatory response, and blood pressure (Nogal et al., 2021). Together, the metabolic capacity of DE111^®^ investigated in this study using genomics, phenotypic and metabolomic analysis further highlights the ability of this *B. subtili*s strain to ferment dietary fibers producing beneficial short-chain fatty acids (SCFAs).

Genes encoding enzymes involved in protein metabolism were also identified in DE111^®^. Proteolysis is the most complex biochemical event that has been extensively studied in Lactic acid bacteria (LAB; *Lactococcus*, *Lactobacillus*, and *Streptococcus*; Savijoki et al., 2006). In LAB, this cascade begins with the activity of a surface proteinase, often called a cell wall, or cell envelope proteinase (CEP). The peptides produced by the activity of CEP are transported into the cell and degraded by the coordinated action of peptidases with different, but often partially overlapping, specificities. This joint activity of peptidases is crucial for achieving release of free amino acids (FAA; Savijoki et al., 2006). Genome analysis of DE111^®^ has revealed the presence of multiple genes encoding cell-enveloped proteinases, encoded by *prtP* and its homologs, responsible for casein hydrolysis. Additionally, presence of CLP ATPases proteases which are reported to be active toward caseins were also identified (Gottesman et al., 1990). In addition, the presence of multiple genes encoding serine proteases could be responsible for the high cleavage of the fluorescently tagged casein derivatives (Figure 4). Together, the high protease activity achieved with DE111^®^ could potentially be a result of the combined action of the caseolytic protease CEP (*prtP*) with the CLP ATPases proteases and serine proteases. The presence of both oligopeptide (OPP) and Dipeptide (DtpP) suggests the ability for oligopeptide and dipeptides uptake and their subsequent metabolism in DE111^®^. The next step after oligo-peptide uptake in proteolysis is the internal hydrolysis of peptides to free amino acids (FAA) by the action of peptidases. There were a diverse range of peptidases identified in the genome of DE111^®^ (Table 3). Further metabolomic analysis confirmed the proteolytic potential of DE111^®^, with a high release of amino acids and free amin acids (FAA) downstream products from milk protein hydrolysis (Figure 5; Supplementary Table S3). Casein-derived bioactive peptides have been shown to exert several positive activities in host affecting the digestive, endocrine, cardiovascular, immune, and nervous systems (McCarthy et al., 2014; Tsakali and Gabriella, 2014; Gillespie and Green, 2016; Chakrabarti et al., 2018). The range of proteases, peptide transporters and peptidases identified in DE111^®^ genome and the release of FAA from the fermented UHT milk suggests that this organism can positively influence on the digestion and utilization of casein, and could potentially generate beneficial casein-derived bioactive peptides. While preliminary genome analysis suggests that this strain is capable of breaking down protein, peptides, carbohydrates and fats, further investigation is required to confirm the expression of such genes which may also play a role in generating beneficial SCFA and bioactive peptides.

Most human diets provide a robust supply of vitamins in addition to gut microbes also contributing to vitamin synthesis. However, the molecular structure of bacterially synthesized vitamins is not always identical to the dietary forms of the vitamins (Morowitz et al., 2011). In fact, a previous study has reported that several specialized epithelial transporters have been recognized to participate specifically in the absorption of vitamins derived from gut bacteria (Morowitz et al., 2011). In this study, DE111^®^ illustrates genetic potential to synthesize water and fat-soluble B-vitamins and vitamin K2 in addition to 5 essential amino acids (threonine, tryptophan, methionine, leucine, and lysine; Table 4). Additionally, antioxidant properties of DE111^®^ were also investigated in this study, genome analysis of DE111^®^ has revealed the presence of multiple genes encoding both catalases (Catalase; AMA54370.1, manganese catalase; AMA51159.1), peroxidases (glutathione peroxidase; AMA52709.1) and other enzymes such as superoxide dismutase (AMA52634.1; not presented in the table).

*In vitro* analysis of total antioxidant activity of DE111^®^ (3178.54 ng/ml) was statically significant than that of the comparator *L. rhamnosus* GG (2034.049 ng/ml). In this sense, consumption of DE111^®^ alone or foods supplemented with DE111^®^ may reduce oxidative damage, free radical scavenging rate, and supply essential vitamins to human body. Altogether, these findings suggest that DE111^®^ could be considered as a potential strain for the preparation of functional foods, offering antioxidant potential and synthesis of SCFAs, bioactive peptides and vitamins.

## 5. Conclusion

This study gives a comprehensive overview of the probiotic attributes of a commercially available *Bacillus subtilis* DE111^®^, starting with whole genome analysis and the identification of key genomic markers attributed to genuine probiotic traits. This is subsequently followed with combined genomic-phenotypic-enzymatic and metabolomic analysis. *In silico* analysis of genes associated with stress tolerance coupled with *in vitro* analysis of DE111^®^ to remain viable at high temperatures (75°C and 90°C) confirmed the thermostability of this strain. Additionally, the presence of several genes associated with gastric stress tolerance identified in this study, correlates well with the previous clinical study (Colom et al., 2021) demonstrating DE111^®^ to remain viable during transit through the stomach and germinate in the small intestine of humans. Further mechanisms present in DE111^®^ that may play a role in positively impacting on host health were investigated using a combination of genomic, enzymatic and metabolic analysis. Results from these analysis indicated a strong potential of DE111^®^ to produce beneficial SCFAs. Additionally, DE111^®^ extensive caseolytic activity highlights potential generation of casein-derived bioactive peptides. DE111^®^ also displayed high antioxidant activity and demonstrates genetic ability to synthesize several B-vitamins, vitamin K2 as well as five essential amino acids (threonine, tryptophan, methionine, leucine, and lysine). Genes encoding several antimicrobial peptides were also identified and *in vitro* analysis of DE111^®^ displayed a broad antimicrobial activity against those that are involved in urinary tract, intestinal and common skin infections suggesting, DE111^®^ has the pottential in controlling or preventing the presence of such opportunistic pathogens. The anti-adhesion potential of DE111^®^ in cell models will be explored in the future, to further understand the protective properties of DE111^®^ toward such pathogens. Together, findings from this study support the ability of DE111^®^ to survive and remain viable under GIT environmental stresses and promote the absorption and utilization of dietary proteins and carbohydrates as well as possibly synthesize SCFA, bioactive peptides, vitamins, and amino acids – all of which confer a health benefit for the host.

## Data availability statement

Publicly available datasets were analyzed in this study. This data can be found here: https://www.ncbi.nlm.nih.gov/nuccore/CP013984.1.

## Author contributions

SM, EK, JC, and AS performed the sample analysis. JD and KR were the advisors for the study and reviewed the manuscript. SM contributed to the writing of the manuscript. All authors contributed to the article and approved the submitted version.

## Funding

This study received funding from Deerland Probiotics and Enzymes.

## Conflict of interest

SM, EK, JC, AS, JD, and KR are employed by Deerland Probiotics and Enzymes.

The authors declare that this study received funding from Deerland Probiotics and Enzymes. The funder was involved in the study design, collection, analysis, interpretation of data, the writing of this article or the decision to submit it for publication.

## Publisher’s note

All claims expressed in this article are solely those of the authors and do not necessarily represent those of their affiliated organizations, or those of the publisher, the editors and the reviewers. Any product that may be evaluated in this article, or claim that may be made by its manufacturer, is not guaranteed or endorsed by the publisher.
