# Supplementary material for: In vitro and in silico assessment of probiotic and functional properties of Bacillus subtilis DE111®
Source: Front Microbiol. 2023 Jan 13;13:1101144. doi: 10.3389/fmicb.2022.1101144 (PMC9880548; doi:10.3389/fmicb.2022.1101144)
Supplement: Supplementary file 1 [file Data_Sheet_1.docx]

Supplementary Material

**Table S1.** Sporulation Proteins identified in DE111^®^ Genome.

| **Sporulation stage** | **Essential sporulation genes present in spore-forming bacilli^a^** | **Genes detected in DE111® genome (Protein Acc Number)** |
| --- | --- | --- |
| Pre-septation | spo0A, sigH (spo0H)b, obgE, spo0F, ytxC | spo0A (AMA52947.1), sigH (AMA50823.1), obgE (AMA53265.1) |
| Post-septation | spoIIAA, spoIIAB, sigF (spoIIAC)b, spoIID, spoIIE (spoIIH), spoIIGA, sigE (spoIIGB), spoIIM, spoIIPa, spoIIR | spoIIPa (AMA53078.1), spoIIB (AMA53329.1), spoIID (AMA54187.1), spoIIR (AMA54209.1), spoIIAA(AMA52872.1), spoIIAB (AMA52871.1), sigE (AMA52223.1), spoIIE (AMA50790.1), spoIIGA (AMA54695.1), spoIIM (AMA52879.1) |
| Post-engulfment | cwlDa, dacB, dapA, dapB, spmA, spmB, spoIIIAA, spoIIIAB, spoIIIAC, spoIIIAD, spoIIIAE, spoIIIAF, spoIIIAG, spoIIIAH, spoIIIDa, spoIIIE, spoIIIJ, sigG (spoIIIG), spoIVA, spoIVB, sigK (spoIIIC+spoIVCB), spoVAC, spoVADa, spoVAEB, spoVB family, pth (spoVC), spoVDa, spoVGa, spoVKa, spoVSa, spoVT, stoA (spoIVH), yabP, yabQa, ylbJ, ylmC, yqfC, yqfD, ytvI, yyaC | SpoIIIAG (AMA52962.1), spoIIIAF(AMA52963.1), spoIIIAH (AMA52961.1), spoIIID (AMA54143.1), spoIIIAE (AMA52964.1), spoIIIAD (AMA54731.1), spoIIIAC (AMA52965.1), spoIIIAA AMA52967.1, spoVAF (AMA52863.1), spoVAEB (AMA52864.1), spoVAE (AMA52865.1), SpoVAD (AMA52866.1), spoVC (AMA52867.1), spoVAB (AMA52868.1), spoVAA(AMA52869.1), spoIVB (AMA52948.1), yabP (AMA50786.1), spoVD (AMA52208.1), spoVM(AMA52270.1), spoVS (AMA52385.1), spoVK (AMA52418.1), spoYPJB (AMA52767.1), spoIIIAB (AMA52966.1), spoVB (AMA53242.1), spoIVFB (AMA53271.1), spoIVFA (AMA53272.1), spoYTFJ(AMA53473.1), cse60(AMA53526.1),YunB(AMA53741.1), YabQ(AMA50787.1), |
| Spore coat formation | spoIVA, alr (yncD), cotA, cotC, cotH, cotI, cotJA, cotJB, cotM, cotP, cotS, cotU, tgl, yisY, yknT | stage IVA (AMA52794.1), cotE (AMA52390.1), cotM (AMA52481.1), ysxE(AMA53334.1), cotNH(AMA54761.1), cotH (AMA54106.1), cotF(AMA54565.1), cotJA (AMA51396.1), cotJB (AMA51397.1), cotJC (AMA51398.1), cotF (AMA51577.1), yheD(AMA51672.1),spaC(AMA51673.1), cotO (AMA51864.1), cotZ (AMA51865.1),cotY (AMA51866.1), cotX(AMA51867.1), cotW (AMA51867.1), cotV (AMA51869.1) and cotT(AMA51896.1) |
| Germination and SASPs | gpr, lgt (gerF), gerA family, gerB family, gerC family, gerM, ypeB, ytgP | lgt (AMA53997.1), ypeB (AMA52806.1), ytgP (AMA52205.1), SleB (AMA52807.1), gerQ(AMA54106.1), SspK (AMA51549.1), SspP (AMA52482.1), SspO(AMA54701.1), SspL(AMA52719.1), SspI (AMA52719.1), SspTlp(AMA52487.1) |

^a^ Sourced from (Galperin et al., 2012; Kapse et al., 2019b)

**Table S2.** Putative genes involved in adhesion and aggregation in DE111^®^ genome.

| **Function** | **Product** | **GenBank Acc Number** |
| --- | --- | --- |
| EPS biosynthesis | poly-gamma-glutamate synthase *pgsBCA* genes | AMA54091.1  AMA54093.1  AMA54092.1 |
|  | Glycosyl transferase, group 1 family protein | AMA54050.1 |
|  | Manganese-dependent protein-tyrosine phosphatase | AMA53594.1 |
|  | protein tyrosine kinase EpsB | AMA53940.1 |
|  | Tyrosine-protein phosphatase YwqE | AMA54122.1 |
|  | Pyruvate carboxylase | AMA52176.1 |
|  | triosephosphate isomerase | AMA53898.1 |
| Flagellum | Flagellar hook-length control protein FliK | AMA52316.1 |
|  | flagellar hook-basal body complex protein FlgG | AMA54140.1 |
|  | flagellar hook-basal body complex protein FliE | AMA52309.1 |
|  | Chitin binding protein | AMA52575.1 |
|  | Enolase | AMA53896.1 |

**Table S3**: List of compounds significantly associated with fermentation of UHT milk by DE111^®^ analyzed with GC-MS. Mean values of compounds identified in DE111^®^ and Control (uninoculated) UHT milk along with the associated potential precursors / peptidases identified in the DE111^®^ genome are presented.

|  |  | **Relative Peak concentration** | | **Putative peptidases/ precursors/ source** |
| --- | --- | --- | --- | --- |
| **Compound** | **P value** | **Mean of Control** | **Mean of DE111** |  |
| Alanine | 0.000016 | 0.04 | 0.4767 | Aminopeptidases (PepN or other Cysteine specific aminopeptidases |
| Glycine | 0.010699 | 0.87 | 3.487 | glycine carboxypeptidase |
| Valine | 0.000333 | 0 | 2.613 | Aminopeptidases (PepN or other Cysteine specific aminopeptidases |
| Leucine | 0.000163 | 0.02 | 1.95 | PepA/ PepN/ Aminopepetidase |
| Isoleucine | 0.000139 | 0 | 2.993 | Aminopeptidases |
| Threonine | 0.002596 | 0.003333 | 8.417 | Aminopeptidases |
| Proline | 0.002485 | 0.1933 | 1.04 | PepP/ PepX |
| Aspartic acid | 0.000998 | -0.2867 | 3.677 | PepE |
| Glutamic acid | 0.015014 | 0.87 | 2.057 | Glutamyl endopeptidase |
| Methionine | 0.000658 | 0 | 4.453 | PepM/ MAP |
| Phenylalanine | 0.001451 | 0 | 3.88 | leucine aminopeptidase |
| Lysine | 0.000636 | 0.01 | 4.643 | PepN/ Aminopepetidase |
| Histidine | 0.000664 | -0.01 | 7.235 | aminopeptidase |
| Tyrosine | 0.001618 | 0 | 4.173 | Aminopeptidase |
| Tryptophan | 0.000361 | 0 | 4.587 | Amiopeptidase |
| Cystine | 0.007182 | -0.01333 | 0.04 | PCP |
| Ornithine | 0.001398 | 0.03667 | 5.153 | Arginine |
| Serine | 0.0045 | 0.27 | 1.807 | seriene peptidase |
| Pyruvic acid | 0.000036 | -0.05667 | 0.09333 | glucose/ alanine |
| Fumaric acid | 0.006877 | -0.4967 | 0.8267 | Maelic acid |
| 2-Oxoglutaric acid | 0.006695 | 0.7567 | 1.087 | Glutamate |
| 4-methyl-2-oxopentanoic acid | 0.00063 | -0.03333 | 0.2 | leucine |
| 3-(methylthio)propionic acid | 0.000848 | 0.04 | 4.227 | Alanine |
| 3-Methyl-oxirane-2-carboxylic acid | 0.000902 | -0.09 | 0.1567 | Amino acid decarboxylation |
| Benzoic acid | 0.003098 | 0.03333 | 1.297 | Phenylalanine |
| Phenylacetic acid | 0.000169 | -0.003333 | 2.257 | Phenylalanine +Glucose |
| Decenoic acid | 0.021611 | 1.333 | 2.46 | Lipolysis of milk fat |
| N-Acetyl-valine | 0.000054 | -0.02 | 4.867 | Valine |
| 4-Hydroxyphenylacetic acid | 0.000265 | 0 | 2.727 | Phenylalanine +Glucose |
| 4-Aminobenzoic acid | 0.000562 | 0 | 0.03333 | Phenylalanine |
| Alanyl-proline | 0.005469 | -0.05667 | 2.473 | PepV |
| Glycyl-proline | 0.002928 | -0.01333 | 5.92 | PepC/ PepD |
|  |  |  |  |  |

**Table S4:** Carbohydrate fermentation profile of DE111^®^ using API 50 CH kit system. All tests were performed in triplicates. All results presented are generated from this study. +, Positive; -, Negative.

|  |  |  |
| --- | --- | --- |
|  | **Carbohydrate** | ***B. subtilis* DE111^®^** |
| 1 | GLYCEROL | + |
| 2 | ERYTHRITOL | - |
| 3 | D-ARABINOSE | - |
| 4 | l-ARABINOSE | + |
| 5 | D-RIBOSE | + |
| 6 | D-XYLOSE | + |
| 7 | L-XYLOSE | - |
| 8 | D-ADONITOL | - |
| 9 | METHYL-BD-XYLOPYRANOSIDE | - |
| 10 | D-GALACTOSE | - |
| 11 | D-GLUCOSE | + |
| 12 | D-FRUCTOSE | + |
| 13 | D-MANNOSE | + |
| 14 | L-SORBOSE | - |
| 15 | L-RHAMNOSE | - |
| 16 | DULCITOL | - |
| 17 | INOSITOL | + |
| 18 | D-MANNITOL | + |
| 19 | D-SORBITOL | + |
| 20 | METHYL-AD-MANNOPYRANOSIDE | - |
| 21 | METHYL-AD-GLUCOPYRANOSIDE | - |
| 22 | N-ACETYLGLUCOSAMINE | - |
| 23 | AMYGDALIN | + |
| 24 | ARBUTIN | + |
| 25 | ESCULIN FERRIC CITRATE | + |
| 26 | SALICIN | + |
| 27 | D-CELLOBIOSE | + |
| 28 | D-MALTOSE | + |
| 29 | D-LACTOSE | - |
| 30 | D-MELIBIOSE | - |
| 31 | D-SACCHAROSE | + |
| 32 | D-TREHALOSE | + |
| 33 | INULIN | - |
| 34 | D-MELEZITOSE | - |
| 35 | D-RAFINOSE | - |
| 36 | AMIDON | - |
| 37 | GLYCOGEN | - |
| 38 | XYLITOL | - |
| 39 | GENTIOBIOSE | - |
| 40 | D-TURANOSE | - |
| 41 | D-LYXOSE | - |
| 42 | D-TAGATOSE | - |
| 43 | D-FUCOSE | - |
| 44 | L-Fucose | - |
| 45 | D-ARABITOL | - |
| 46 | L-ARABITOL | - |
| 47 | POTTASIUM gLUCOnATE | - |
| 48 | POTTASIUM 2-KETOGLUCONATE | - |
| 49 | POTTASIUM 5-Ketogluconate | - |

**Table S5.** Semi-Quantitative assay of enzyme activities of DE111^®^. All tests were performed in triplicates. All results presented are generated from this study.

|  | | **DE111^®^** |
| --- | --- | --- |
| Esterase activity | Esterase (C4:0) | + |
|  | Esterase (C8:0) | + |
| Lipase activity | Lipase (C14:0) | - |
| Peptidase activity | Leucine arylamidase | - |
|  | Valine arylamidase | - |
|  | Cystine arylamidase | - |
| Proteinase activity | Trypsin | - |
|  | α-chymotrypsin | - |
| Phosphatase activity | Acid phosphatase | + |
|  | Alkaline phosphatase | + |
|  | Phosphohydrolyase | + |
| Glycosidase activity | α-Galactosidase | - |
|  | β-Galactosidase | - |
|  | β-Glucuronidase | - |
|  | α-Glucosidase | + |
|  | β-Glucosidase | + |
|  | β-Glucosaminidase | - |
|  | α-Mannosidase | - |
|  | α-Fucosidase | - |
| Color shading shows green (positive) and red (negative). | |  |

**
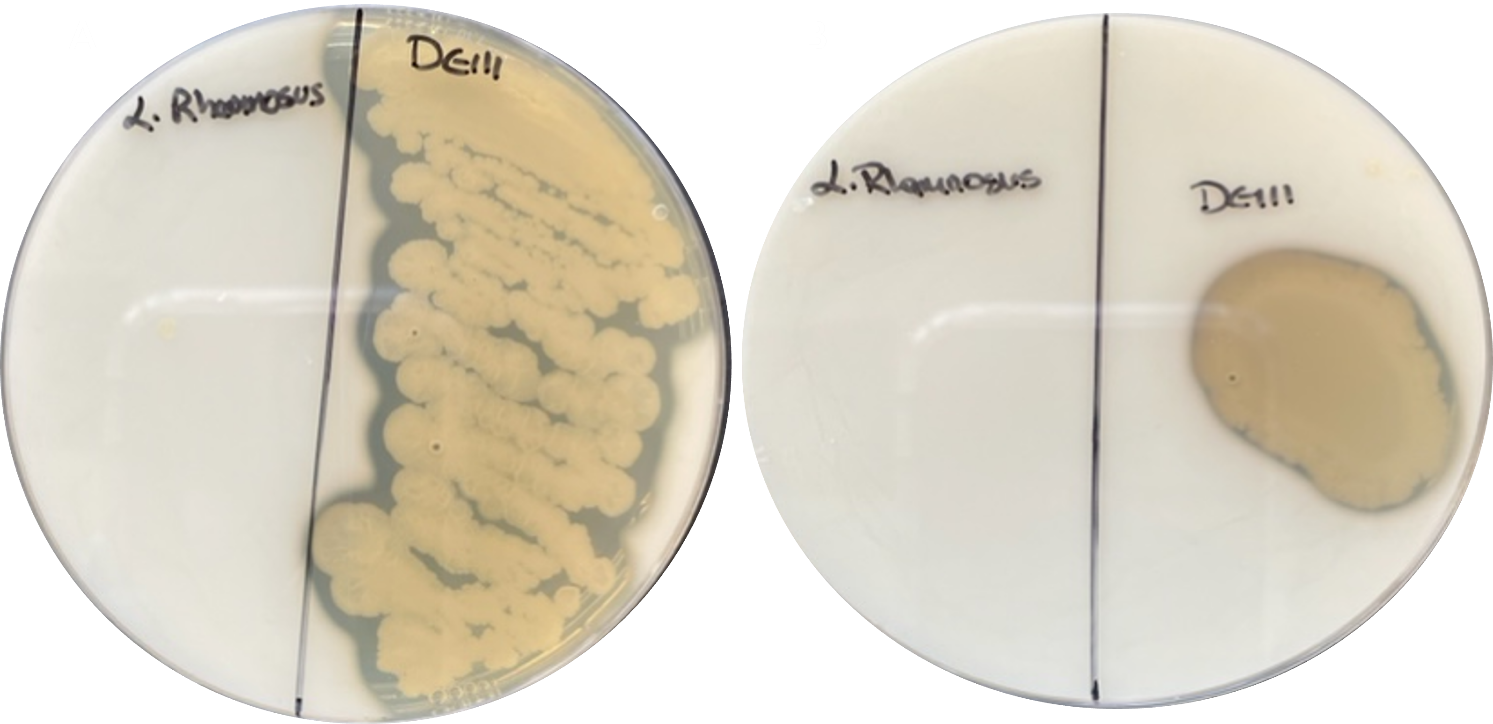
SUPPLEMENTARY FIGURE**

**Figure S1.** High caseolytic activity of DE111^®^ on reconstituted skim milk agar (RSM) after 24 h incubation at 37⁰C. Activity assessed by (a) streaking DE111^®^ (right) and comparator probiotic strain *L. rhamnosus* GG (left), and (b) spotting DE111^®^ (right) and comparator probiotic strain *L. rhamnosus* GG on RSM agar (left).


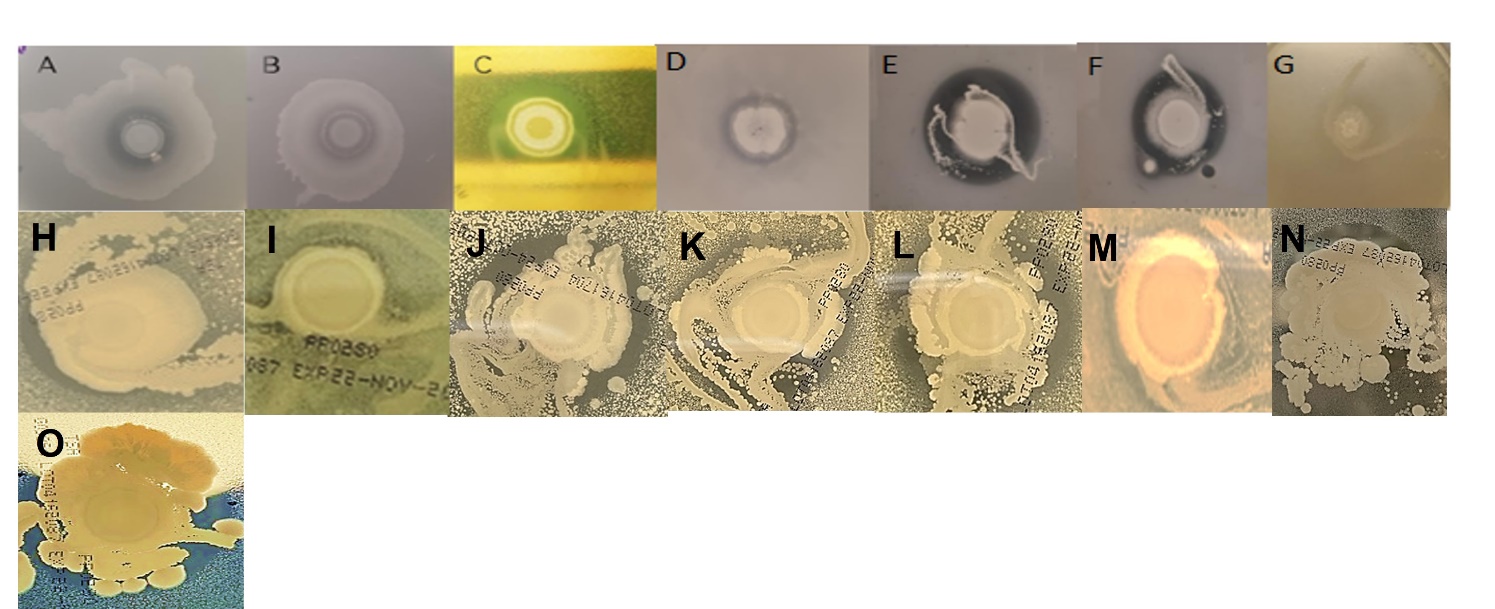

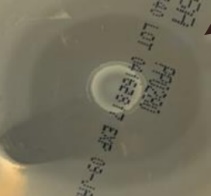


**P**


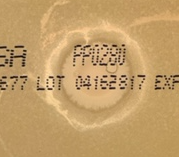


**Q**


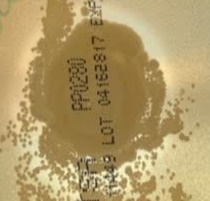


**R**

**Figure S2.** Antimicrobial activity of DE111^®^ against gut, skin, and urinary tract opportunistic pathogens. (A) *Escherichia coli* ATCC25922, (B) *Salmonella enterica* ATCC13076, (C) *Pseudomonas aeruginosa* DSM3227 *,* (D) *Staphylococcus aureus* DSM17091, (E) *Staphylococcus epidermidis* DSM20044, (F) *Staphylococcus warneri* DSM20316*,* (G) *Cutibacterium acnes* DSM1897, (H) *Shigella flexnerii* DSM4782, (I) *Candida albicans* DSM3454, (J) *Staphylococcus pseudintermedius* DSM21284, (K*) Staphylococcus saprophyticus* DSM20229, (L) *Staphylococcus aureus* DSM1104, (M) *Acinetobacter baumannii* DSM30007, (N) *Streptococcus agalactiae* DSM2134, (O) *Streptococcus pyogenes* DSM20565, (P) *Gardernella vaginalis* DSM4944, (Q) *Bacillus cereus* DSM31, and (R) *Campilobacter jejuni* DSM4688.
